# Identification of Key Residues for Enzymatic Carboxylate Reduction

**DOI:** 10.3389/fmicb.2018.00250

**Published:** 2018-02-19

**Authors:** Holly Stolterfoht, Georg Steinkellner, Daniel Schwendenwein, Tea Pavkov-Keller, Karl Gruber, Margit Winkler

**Affiliations:** ^1^Institute of Molecular Biotechnology, Graz University of Technology, NAWI Graz, Graz, Austria; ^2^Austrian Centre of Industrial Biotechnology, Graz, Austria; ^3^Structural Biology, Institute of Molecular Biosciences, University of Graz, Graz, Austria

**Keywords:** carboxylate reductase, biocatalysis, signature sequence, aldehyde, flavor and fragrance, pharmaceutical intermediate

## Abstract

Carboxylate reductases (CARs, E.C. 1.2.1.30) generate aldehydes from their corresponding carboxylic acid with high selectivity. Little is known about the structure of CARs and their catalytically important amino acid residues. The identification of key residues for carboxylate reduction provides a starting point to gain deeper understanding of enzymatic carboxylate reduction. A multiple sequence alignment of CARs with confirmed activity recently identified in our lab and from the literature revealed a fingerprint of conserved amino acids. We studied the function of conserved residues by multiple sequence alignments and mutational replacements of these residues. In this study, single-site alanine variants of *Neurospora crassa* CAR were investigated to determine the contribution of conserved residues to the function, expressability or stability of the enzyme. The effect of amino acid replacements was investigated by analyzing enzymatic activity of the variants *in vivo* and *in vitro*. Supported by molecular modeling, we interpreted that five of these residues are essential for catalytic activity, or substrate and co-substrate binding. We identified amino acid residues having significant impact on CAR activity. Replacement of His 237, Glu 433, Ser 595, Tyr 844, and Lys 848 by Ala abolish CAR activity, indicating their key role in acid reduction. These results may assist in the functional annotation of CAR coding genes in genomic databases. While some other conserved residues decreased activity or had no significant impact, four residues increased the specific activity of *Nc*CAR variants when replaced by alanine. Finally, we showed that *Nc*CAR wild-type and mutants efficiently reduce aliphatic acids.

## Introduction

Aldehydes are widely used as flavors and fragrances (Hagedorn and Kaphammer, 1994; Lesage-Meessen et al., 1996; Thibault et al., 1998; Kunjapur et al., 2014), chemicals and precursors for pharmaceuticals (Tripathi et al., 1997). Furthermore, they are precursors for fatty alcohols, which, in turn, can be used as biofuels (Akhtar et al., 2013), polymer constituents, surfactants, detergents, and cosmetics (Noweck and Grafahrend, 2006). Enzymatic carboxylate reduction is catalyzed either by ATP/NADPH dependent proteins of the family E.C. 1.2.1.30 [originally termed aryl-aldehyde dehydrogenase (NADP^+^); now known as carboxylate reductase (CAR)] or by molybdenum or tungsten dependent proteins of the family E.C. 1.2.99.6 (termed aldehyde:ferredoxin oxidoreductases or also carboxylate reductase) (White et al., 1989, 1993; Mukund and Adams, 1991; Huber et al., 1994; Ni et al., 2012). Carboxylate reductases (E.C. 1.2.1.30) are able to generate various aldehydes with high selectivity from their corresponding carboxylic acid, which can be available from natural and renewable resources (Yang et al., 2007). Generally, enzymatic carboxylate reduction provides several advantages compared to chemical reductions: The reactions are carried out in one step under mild conditions in aqueous solutions. CARs accept a broad range of substrates, while being product-selective (no overreduction of the aldehydes to the respective alcohols), chemo-selective (no reduction of other reducible moieties), and to some degree enantioselective (Napora-Wijata et al., 2014; Qu et al., 2018; Winkler, 2018).

Due to these characteristics and the growing demand for bio-based products from renewable resources, CARs are gaining attention as biocatalysts for chemical synthesis.

However, only a few enzyme sequences have been identified and characterized to date (Table 1). The limited number of known enzymes is surprising in light of the plethora of fungi and bacteria that can perform carboxylate reduction (Napora-Wijata et al., 2014).

**Table 1:** Known CAR enzymes that have been characterized to date.

**Entry**	**Enzyme Abbrev**.	**Organism**	**Accession No**.	**Phylogenetic group**	**References**
1	*Ni*CAR	*Nocardia iowensis*	Q6RKB1.1	type I	Li and Rosazza, 1997; He et al., 2004
2	*Ms*CAR2	*Mycobacterium smegmatis* str. MC2 155	YP_889972.1	type I	Hu, 2010; Khusnutdinova et al., 2017
3	*Mt*CAR	*Mycobacterium tuberculosis*	NP_217106.1	type I	Hu, 2010
4	*Msp*CAR1	*Mycobacterium* sp. JLS	WP_011855500.1	type I	Behrouzian et al., 2010
5	*Mm*CAR	*Mycobacterium marinum*	WP_012393886.1	type I	Akhtar et al., 2013
6	*Ms*CAR1	*Mycobacterium smegmatis* str. MC2 155	YP_887275.1	type I	Schaffer et al., 2014; Khusnutdinova et al., 2017
7	*Sro*CAR	*Segniliparus rotundus*	WP_013138593.1	type I	Duan et al., 2015a
8	*Mn*CAR	*Mycobacterium* sp. *(neoaurum)*	WP_019510583.1	type I	Duan et al., 2015b
9	*Msp*CAR2	*Mycobacterium* sp. JS623	WP_015306631.1	type I	Moura et al., 2016
10	*Nb*CAR1	*Nocardia brasiliensis*	AFU02004.1	type I	Moura et al., 2016
11	*Mp*CAR	*Mycobacterium phlei*	WP_003889896.1	type I	Finnigan et al., 2017
12	*Ms*CAR3	*Mycobacterium smegmatis* str. MC2 155	AFP42026.1	type I	Finnigan et al., 2017
13	*No*CAR	*Nocardia otitidiscaviarum*	WP_029928026.1	type I	Finnigan et al., 2017
14	*Tp*CAR	*Tsukamurella paurometabola*	WP_013126039.1	type I	Finnigan et al., 2017
15	*Sru*CAR	*Segniliparus rugosus*	WP_007468889.1	type I	Gahloth et al., 2017
16	*Ma*CAR1	*Mycobacterium abscessus* ATCC 19977	CAM63040.1	type I	Khusnutdinova et al., 2017
17	*Ma*CAR2	*Mycobacterium abscessus* ATCC 19977	CAM64782.1	type I	Khusnutdinova et al., 2017
18	*Map*CAR	*Mycobacterium avium* subsp. *paratuberculosis*	AAS03357	type I	Khusnutdinova et al., 2017
19	*Mc*CAR	*Mycobacterium chelonae*	AKC40871.1	type I	Khusnutdinova et al., 2017
20	*Mi*CAR1	*Mycobacterium immunogenum*	KPG37443.1	type I	Khusnutdinova et al., 2017
21	*Mi*CAR2	*Mycobacterium immunogenum*	KPG36834.1	type I	Khusnutdinova et al., 2017
22	*Nb*CAR2	*Nocardia brasiliensis*	GAJ83027	type I	Khusnutdinova et al., 2017
23	*At*CAR	*Aspergillus terreus*	XP_001212808.1	type II	Wang and Zhao, 2014; Wang et al., 2014
24	*Sb*CAR	*Stachybotrys bisbyi*	BAV19380.1	type II	Li et al., 2016
25	*Nc*CAR	*Neurospora crassa*	XP_955820.1	type III	Gross et al., 1968; Schwendenwein et al., 2016
26	*Tv*CAR	*Trametes versicolor*	XP_008043822.1	type IV	Winkler and Winkler, 2016

CARs show significant similarities to non-ribosomal peptide synthases (NRPS), members of the ANL (Acyl-CoA synthetase/NRPS adenylation domain/Luciferase) superfamily of adenylating enzymes (Gulick, 2009), as they are comprised of an *N*-terminal adenylation domain (A-domain), a phosphopantetheine attachment site, also called transthiolation domain (T-domain), or peptidyl carrier protein (PCP) domain, and a *C*-terminal reductase domain (R-domain). On the molecular level, a cascade reaction proceeds: First, deprotonated carboxylic acid is activated as adenosyl monophosphate at the expense of ATP at the A-domain. The AMP-intermediate is nucleophilically attacked by sulfhydryl of a phosphopantetheinyl moiety and, with the release of AMP, a covalently bound thioester is formed. Finally, the thioester is reduced by NADPH to release the aldehyde product at the R-domain (Gross, 1972; Li and Rosazza, 1997).

To obtain active enzyme, CARs require a post-translational phosphopantetheinylation catalyzed by a phosphopantetheine transferase (PPTase), that transfers a phosphopantetheine moiety from coenzym A to a conserved serine residue of apo-CAR (Venkitasubramanian et al., 2007), which provides the sulfhydryl group mentioned above. In addition, like other ATP dependent enzymes, CARs are dependent on Mg^2+^ (Gross and Zenk, 1969).

Recently, first crystal structures of the individual (di)domains of *Sru*CAR, the A-domain of *Ni*CAR, and R-domain of *Mm*CAR shed some light on the complex mechanism underlying catalysis and revealed domain movements during the different steps of the reaction (Gahloth et al., 2017).

The *N*-terminus of CARs shows high homology to known AMP-binding proteins with a similar reaction mechanism to that of the ANL superfamily of adenylating enzymes, whereas the *C*-terminal domain strongly resembles NADPH-binding proteins, like terminal reductase domains of NRPS or short-chain dehydrogenases/reductases (SDRs). A-domains of NRPS enzymes have been studied in detail (Marahiel et al., 1997), key residues identified, and selectivity conferring codes of these highly substrate specific A-domains have been defined (Stachelhaus et al., 1999; Bushley et al., 2008). For SDRs, conserved sequence motifs, and functionally important residues have been extensively characterized as well (Persson et al., 2003; Kavanagh et al., 2008; Chhabra et al., 2012), but CARs are much less thoroughly studied in this regard.

In this study, we used a CAR from the ascomycete *Neurospora crassa* (*Nc*CAR). *Nc*CAR was first isolated from the fungus (strain SY7A) in 1968 (Gross et al., 1968). Notably, the overall domain organization of *Nc*CAR resembles that of type I bacterial CARs, however, sequence identities are very low (~20%) and bacterial CARs are characterized by an A-domain stretch that is not found in type II-IV CARs (Stolterfoht et al., 2017). Consequently, detailed structural information is not easily transferable from one subtype to the other.

The substrate scope of *Nc*CAR determined in the 1970s led to the historic classification of this enzyme as an aryl-aldehyde:NADP oxidoreductase. Recently, the genome of *N. crassa* OR74A was sequenced (Galagan et al., 2003) and we identified the hypothetical protein with the NCBI accession no. XP_955820.1 from this strain to have CAR activity. The heterologously expressed enzyme was further characterized and used for the preparative scale synthesis of the aromatic aldehyde piperonal (Schwendenwein et al., 2016). In contrast to the observations concerning the substrate scope of *Nc*CAR (Gross and Zenk, 1969; Gross, 1972), we show herein that recombinant *Nc*CAR is not restricted to aromatic acids: *Nc*CAR can also reduce aliphatic acids.

## Materials and methods

### Chemicals and solvents

ATP was obtained from Sigma-Aldrich. NADPH, MES, and MgCl_2_ hexahydrate were purchased from Roth. All other chemicals were obtained from Sigma-Aldrich/Fluka or Roth and used without further purification.

### Multiple sequence alignments

Twenty-six confirmed CAR sequences that have been characterized to date (Table 1) were aligned using T-Coffee (http://www.tcoffee.org) as well as ClustalW (http://www.genome.jp/tools-bin/clustalw) with a gap opening penalty of 25 and gap extension penalty of 0.2. The alignment programs were run on the command line and visualized using Jalview (http://www.jalview.org). Additionally, alignments were generated using the program CLC Main Workbench Alignment with default settings. Only slight differences between the alignment tools were encountered, whereas highly conserved fractions of sequences were identical, independent of the alignment program. Conserved sequences presented in Table 2 are based on alignments using T-Coffee.

**Table 2:**
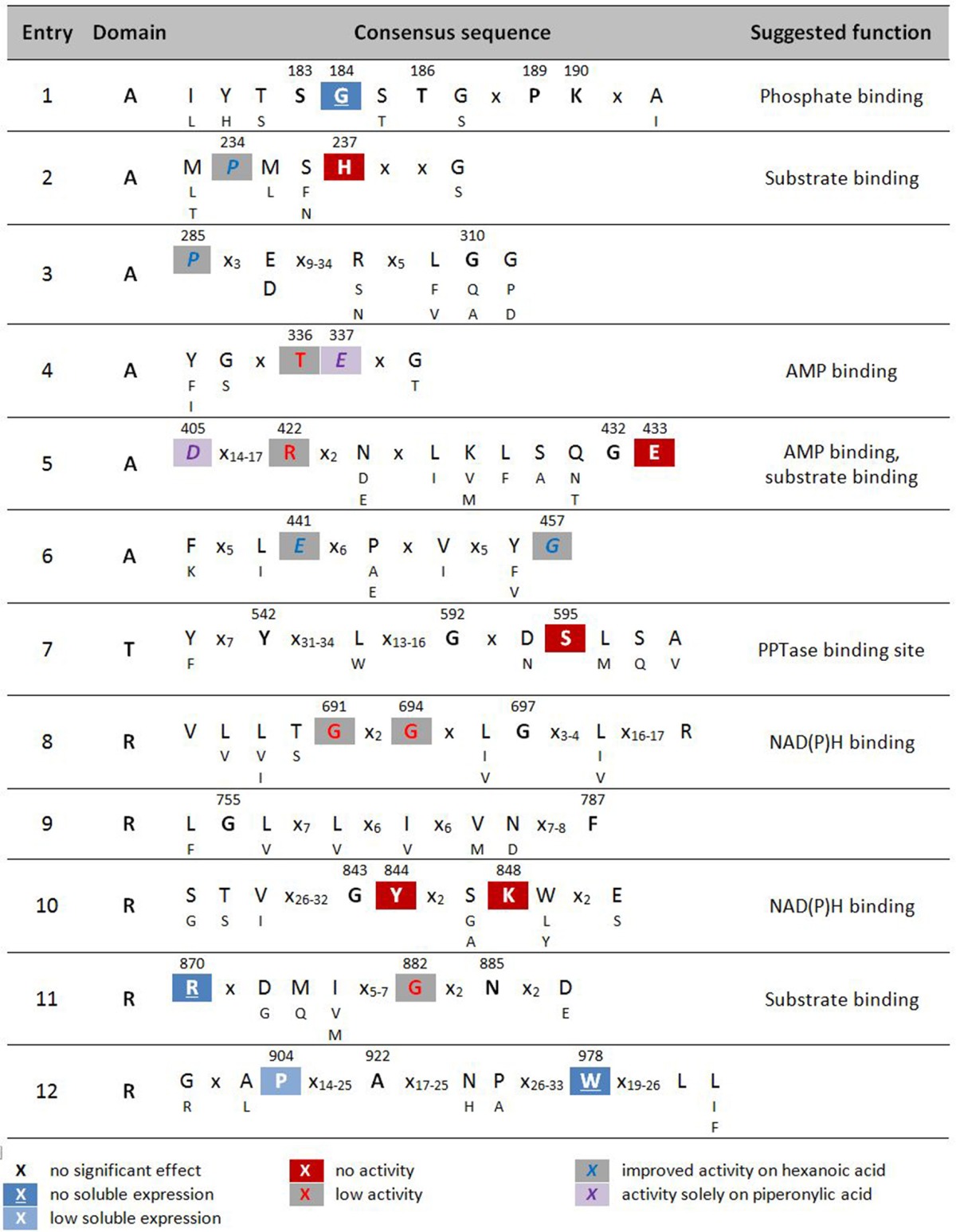
Highly conserved signature sequences of CARs and summary of results of alanine scan of *Nc*CAR.

*Bold amino acids were exchanged in the mutagenesis study (positions of residues are numbered according to NcCAR)*.

X no significant effect [-6,-4]red18pt12ptX no activity [-6,-4]gray18pt12ptX improved activity on hexanoic acid

### Modeling of *Nc*CAR-FL

The program YASARA structure (v. 16.6.24, Krieger et al., 2002) was used to build the models of the A-T-didomain and the full length *Nc*CAR (*Nc*CAR-FL). The target sequence was used to perform a BLAST (Basic Local Alignment Search Tool) search against the UniProt database (PS-BLST iterations 1, *E*-value cutoff 0.01) to build position-specific scoring matrices (PSSM) from related sequences. This profile was used to search the Protein Database for potential modeling templates. Common protein purification tags were thereby excluded. The templates were ranked based on the alignment score and the structural quality. The *Nc*CAR model was built using the top 10 scoring templates with a maximum oligomeric state setting of 1. None of the newly available CAR structures (Gahloth et al., 2017) were on this list as they share lower sequence identities and alignment scores to the *Nc*CAR sequence than the templates which were finally used. For each available template, the alignment with the target sequence was obtained using sequence-based profiles of target and templates calculated from related UniProt sequences. Ligands present in the template structures were parameterized and included in the homology modeling process. Side-chain rotamers were generated and accepted or rejected based on a repulsive energy function.

Loops were optimized using different conformations and side-chain re-optimizations (maximum of 50 conformations per loop). Side-chain rotamers were fine-tuned considering electrostatic and knowledge-based packing interactions as well as solvation effects. The hydrogen bonding network was optimized, including possible ligands. After that, an unrestrained high-resolution refinement with explicit solvent molecules was performed, using knowledge-based force fields. The result was validated to ensure that the refinement did not move the model in a wrong direction. A hybrid model was built out of the top scoring models, where bad regions in the model were iteratively replaced with corresponding fragments from other models.

The final hybrid model of *Nc*CAR-FL is mainly based on PDB structure 5JRH (Han et al., 2016) which was used as template for the assumed A-domain of *Nc*CAR (433 residues aligned, 22% sequence identity). PDB entry 4W4T (Barajas et al., 2015) was used for modeling the assumed R-domain (321 residues aligned, 31% sequence identity) and entry 4DOW (Kuo et al., 2012) was used to model parts of the assumed A-T-didomain (311 residues aligned, 26% sequence identity). Most of the found A-domain templates were acetyl-coenzyme A related enzymes which have approximately the same fold as A-domains of CARs. We also created a full-length model of *Nc*CAR (*Nc*CAR-FL) based on structures of CAR from *Segniliparus rugosus* (*Sru*CAR, PDB codes 5MSW, and 5MSV) determined recently (Gahloth et al., 2017). In order to obtain a full length template to build the *Nc*CAR-FL model on, we first created a fusion model based on the crystal structures of *Sru*CAR (UniProt: E5XP76) where the A-T-didomain was in an “open” position and the T part in the structure of the R-domain was present. The A-T and the T-R parts were structurally aligned and connected. This “fusion” template was then energy minimized using the YASARA standard energy minimization protocol to remove remaining clashes formed by the overlay of the two crystal structures, resulting in a full length *Sru*CAR model similar to the model obtained by Gahloth et al. (2017). Based on this template, the *Nc*CAR-FL model was created using the YASARA homology model protocol restricting it to use the *Sru*CAR full length template. We also created models of the A-domain of *Nc*CAR using the “closed” structure of the A-T-didomain (PDB code 5MSS) as a template. In this “closed” template, the T-domain is in close contact to the A-domain, whereas in the “open” template, the T-domain is flipped away from the A-domain. Again, sequence identities were low, about 21% (793 residues aligned) over the full length *Nc*CAR and around 20% (505 residues aligned) using the “closed” template of the A-T-didomain of *Sru*CAR. As the two models of the full length *Nc*CAR were built using different templates, especially the T part consists of different conformations and the models were not aligned directly. Therefore, to compare the two models we aligned the A-domain and the R-domain separately. The R-domains of the two *Nc*CAR-FL models (313 residues aligned) have a Cα- RMSD of 3.2 Å and the A-domains (365 residues aligned) a Cα- RMSD of 4.6 Å.

### Site-directed mutagenesis

Seventeen Mutants of the *Nc*CAR gene were constructed as described previously (Stolterfoht et al., 2017). Another 17 *Nc*CAR mutants were generated by site-directed mutagenesis according to Agilent's QuikChange II protocol using *Pfu*Ultra High-Fidelity DNA Polymerase (Agilent Technologies). The pETDUET1_*Ec*PPTaseHT*Nc*CAR plasmid was used as the template. Primers were ordered at Integrated DNA Technologies, see Supplementary Information (Table S1). PCR was carried out under the following conditions: 50 μL total volume, 200 μM of each dNTP, 0.2 μM of each primer, 5 ng of template plasmid DNA, and 2.5 U of *Pfu*Ultra High-Fidelity DNA polymerase. Cycling conditions were as follows: initial denaturation at 95°C for 2 min, followed by 16 cycles of denaturation at 95°C for 30 s, annealing at 61°C for 30 s, extension at 72°C for 11 min, and a final extension at 72°C for 15 min. Amplification was controlled by electrophoresis of 10 μL of the PCR reaction on an agarose gel. After the digestion of parental non-mutated methylated plasmid DNA with *Dpn*I at 37°C for 1 h, the samples were desalted and electro-competent *Escherichia coli* Top10F′ cells were transformed with the resulting nicked vector DNA containing the desired mutations.

Plasmid DNA from transformants was checked by restriction site analysis using *Xba*I, *Hind*III, *EcoR*V, *Xho*I, and the correct mutation was confirmed by automated sequencing (Microsynth).

### Protein expression and purification

**Figure 1:**
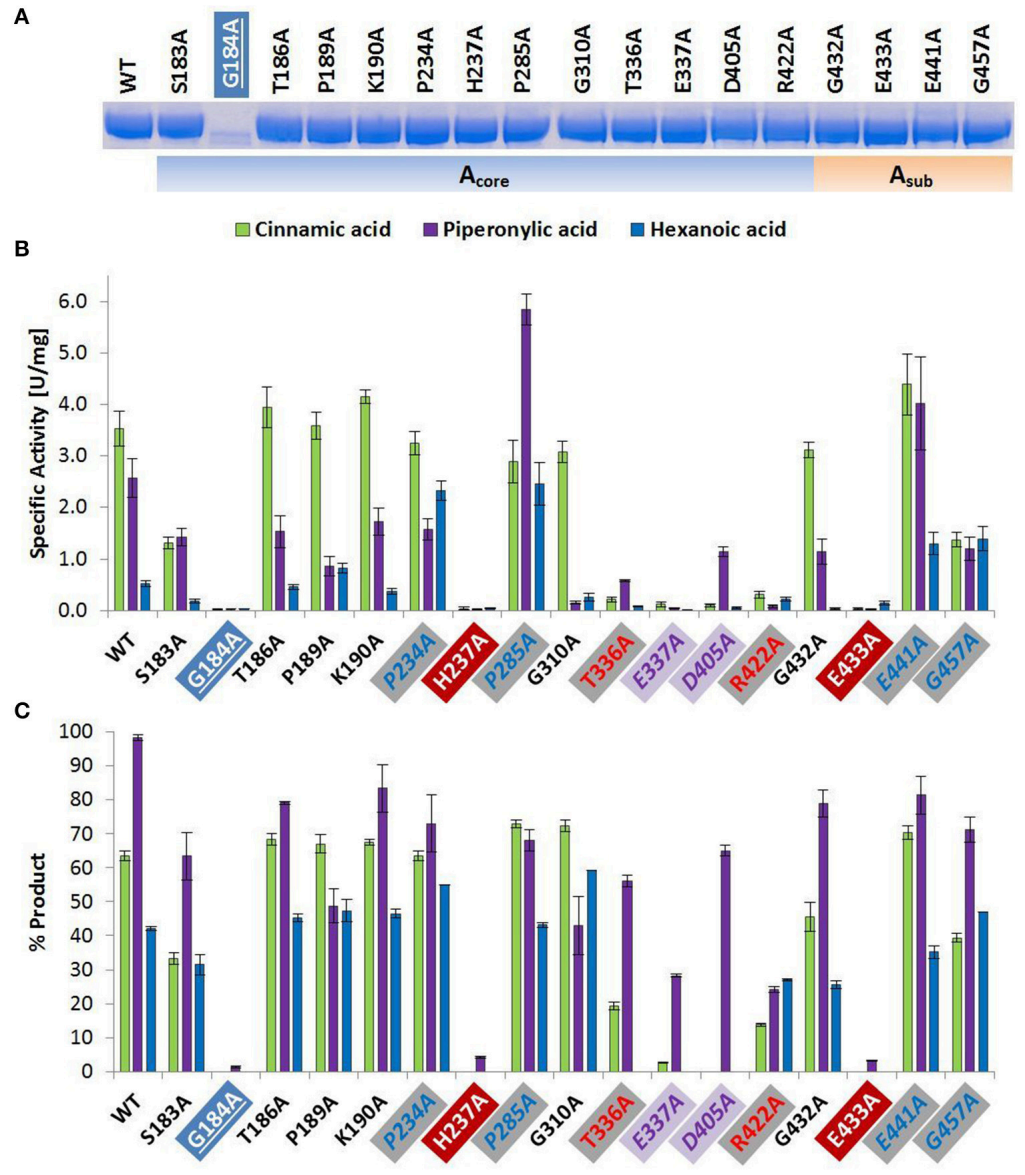
Wild type and alanine mutants of *Nc*CAR A_core_- and A_sub_-domain **(A)** SDS-PAGE of Ni-affinity chromatography purified wild-type enzyme and 17 *Nc*CAR variants (protein amount loaded: 10 μg). For the full gel see Supporting Information Figure S1 **(B)** NADPH consumption was followed at 340 nm and 28°C for 10 min. Data are mean values of three separate experiments, each carried out in four technical replicates. **(C)** Result of whole cell biotransformation after 3 h at 28°C, shown as mean value of triplicates. Cinnamic acid and piperonylic acid reduction was analyzed by HPLC (UV = 254 nm). Green bars represent the sum of cinnamaldehyde and cinnamic alcohol, and violet bars piperonal, respectively. Hexanoic acid reduction was analyzed by GC-FID. Blue bars represent the sum of hexanal and hexanol. For the legend of highlights see Table 2 footnotes.

**Figure 2:**
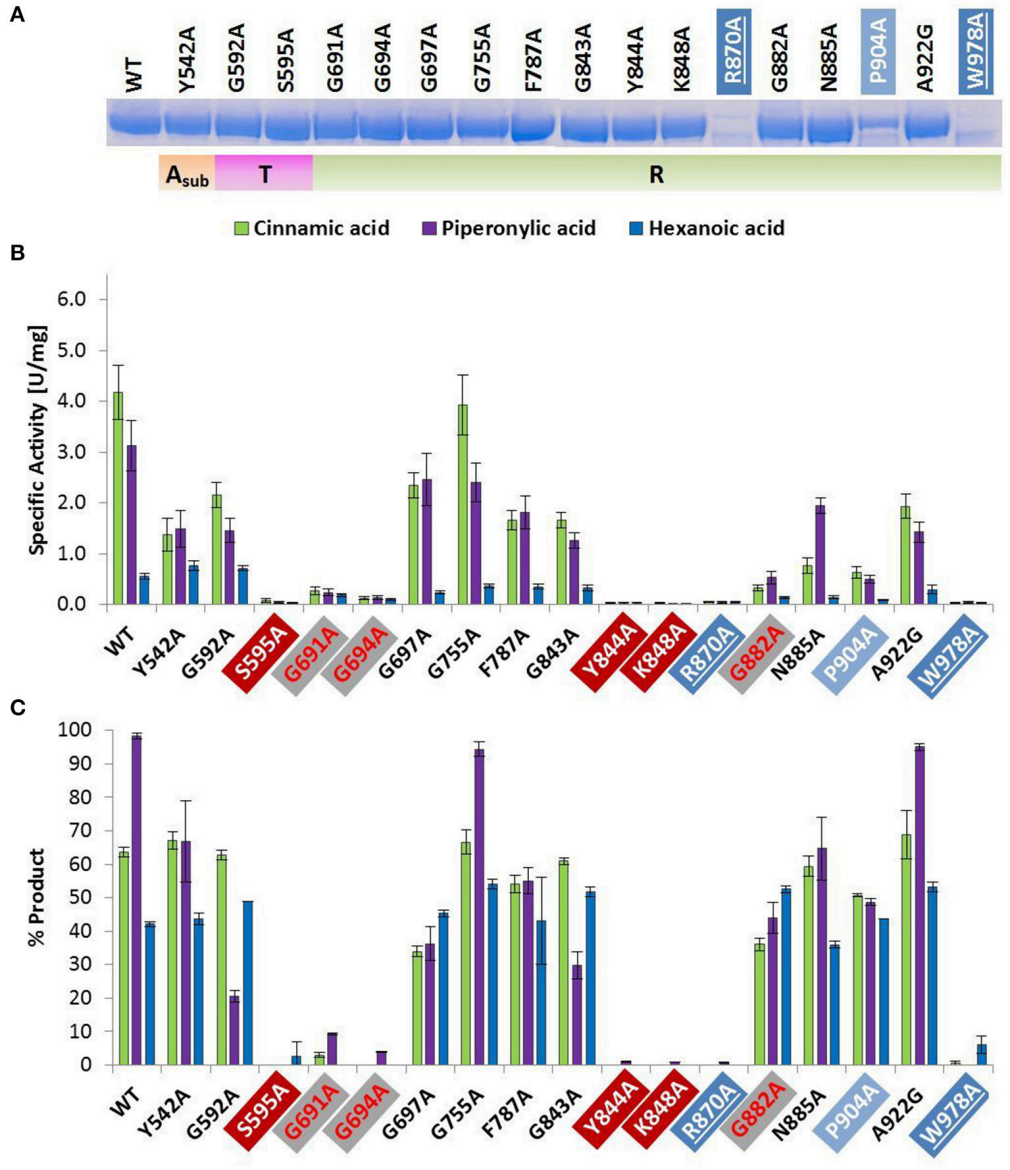
Wild type and alanine mutants of *Nc*CAR A_sub_-, T-, and R-domain **(A)** SDS-PAGE of Ni-affinity chromatography purified wild-type enzyme and 17 *Nc*CAR variants (protein amount loaded: 10 μg). For the full gel see Supporting Information Figure S1 **(B)** NADPH consumption was followed at 340 nm and 28°C for 10 min. Data are mean values of three separate experiments, each carried out in four technical replicates. **(C)** Result of whole cell biotransformation after 3 h at 28°C, shown as mean value of triplicates. Cinnamic acid and piperonylic acid reduction was analyzed by HPLC (UV = 254 nm). Green bars represent the sum of cinnamaldehyde and cinnamic alcohol, and violet bars piperonal, respectively. Hexanoic acid reduction was analyzed by GC-FID. Blue bars represent the sum of hexanal and hexanol. For the legend of highlights see Table 2 footnotes.

*E. coli* K12 MG1655 RARE [for reduced aldehyde reduction, a strain in which seven genes encoding enzymes with confirmed activity on benzaldehyde were deleted (Kunjapur et al., 2014)] was transformed with the pETDUET1_*Ec*PPTaseHT*Nc*CAR wild-type and mutant vectors, and colonies were selected on LB/ampicillin (Amp). For protein expression, the autoinduction protocol as described by Studier was used (Studier, 2005). Cultivations were performed in HT Multitron shakers (Infors AG). After 4 h at 37°C and 24 h at 20°C, the cells were harvested with an Avanti J-20 XP centrifuge (Beckman Coulter), and stored at −20°C until further use. Thawed cells were resuspended in binding buffer (20 mM KP_i_ pH 7.4, 0.5 M NaCl, 10 mM imidazole), disrupted by sonication using a 102C converter with a Sonifier 250 (Branson), and the cell-free extract (CFE) was obtained by centrifugation at 20,000 × g, 4°C, for 1 h. The pellet was used to visualize CAR in insoluble fractions (IFs). Therefore, the pellet was resuspended in 6 M urea and further thoroughly dissolved by heating to 60°C for 45 min with vortexing in between. 1 mL was transferred to a microcentrifuge tube and centrifuged for 30 min at 16,000 × g and 4°C. The CFE was used for protein purification by nickel affinity chromatography on Ni Sepharose™ 6 Fast Flow (GE Healthcare) using the gravity flow protocol. The protein containing fractions were pooled and the buffer was exchanged for 50 mM MES buffer, pH 7.5, containing 10 mM MgCl_2_, 1 mM EDTA, and 1 mM DTT by size exclusion chromatography using PD-10 columns (GE-Healthcare) with the gravity flow protocol. Protein concentrations of the CFEs, IFs, and Ni-affinity purified proteins were determined with the Pierce™ BCA Protein Assay Kit (Thermo Scientific) using BSA as standard. Aliquots of the purified protein solutions were shock frozen in liquid nitrogen and stored at −80°C. For SDS-PAGE of the purified protein samples (Figure S1, Figures 1A, 2A), 10 μg of protein were denatured with NuPAGE™ LDS Sample Buffer for 10 min at 80°C and SDS-PAGE was performed using Novex® 4–12% Bis-Tris-gels (50 min at 200 V and 120 mA in MOPS-buffer), followed by staining using SimplyBlue™ SafeStain (Invitrogen). The same procedure was performed for SDS-PAGE of the CFEs (Figure S2). For SDS-PAGE of the IFs (Figure S3), NuPAGE™ Sample Reducing Agent (Thermo Scientific), and NuPAGE™ LDS Sample Buffer were added to the protein sample for denaturation.

### Spectrophotometric assay

The activity of *Nc*CAR variants compared to the wild-type toward three different substrates was determined monitoring NADPH depletion. The assay composition was as follows: 10 μL of NADPH (10 mM in water), 10 μL of ATP (20 mM in water), 10 μL of CAR enzyme preparation from Ni-affinity chromatography, 160 μL of MES buffer (50 mM, pH 6.0, containing 10 mM MgCl_2_), and 10 μL of 100 mM substrate (cinnamic acid, piperonylic acid, or hexanoic acid) in 0.1 M KOH. Enzymes with little or no activity were used without further dilution (0.6–3.3 mg/mL). Active enzymes were used in appropriate dilutions (≥0.15 mg/mL). The depletion of NADPH was followed on a Synergy Mx Platereader (BioTek) at 340 nm and 28°C for 10 min. Blank reactions without ATP were carried out in parallel. For each protein sample, at least three separate experiments were carried out in four technical replicates, respectively.

### Whole cell biotransformations

The pETDuet1_*Ec*PPTaseHT*Nc*CAR vectors carrying the wild-type and the 34 mutant genes were transformed into electrocompetent *E. coli* K-12 MG1655 RARE cells. Overnight cultures in LB-0.8G medium (LB medium containing 0.8% glucose, 25 mM (NH_4_)_2_SO_4_, 50 mM KH_2_PO_4_, 50 mM Na_2_HPO_4_, 1 mM MgSO_4_, and 100 μg/mL Ampicillin) were inoculated with a single colony, and 2 μL were used to inoculate 100 μL of LB-5052 medium (LB-0.8G medium but with 0.5% glycerol, 0.2% lactose, and 0.05% glucose instead of 0.8% glucose) in 96-well microtiter plates (MTPs). After glucose depletion (6 h of growth at 37°C, 600 rpm, in an Eppendorf Thermomixer), the temperature was lowered to 20°C for protein expression. After 24 h, 100 μL of reaction mixture were added directly to the cell suspensions in the MTP, and sealed with an adhesive seal. The reactions were carried out in triplicates (three wells for each variant/wild-type) in a total volume of 200 μL containing 50 mM potassium phosphate buffer, pH 7.4, 0.12 M glucose, 21 mM citrate, 4 mM MgSO_4_, and 4 mM cinnamic/piperonylic acid, or 3 mM hexanoic acid, at 28°C, 600 rpm. The cinnamic and piperonylic acid conversions were stopped after 3 h by addition of methanol 1:1 v/v and used for HPLC-UV analysis after removal of biomass by centrifugation. Reactions with hexanoic acid as the substrate were frozen at −80°C before further sample preparation for GC-FID analysis. After thawing, 5 μL of 3 M HCl were added to each well to protonate hexanoic acid, as well as 95 μL of ddH_2_O. 250 μL of each well were transferred to a microcentrifuge tube and extracted into 250 μL of ethyl acetate supplemented with 0.01% tetradecane as internal standard, vortexed, and centrifuged for 2 min at 16,000 × g. The organic phase was dried with sodium sulfate (5–10% v/v), and the supernatant was analyzed by GC-FID. The final substrate/product concentration was 2 mM in all samples.

### Chromatographic analyses

For substrate and product quantification by HPLC, reference acids, aldehydes, and alcohols were measured in different concentrations (0.050–2.5 mM), respectively, to calculate standard curves for each compound. Concentrations were calculated using the linear interpolation. The HPLC-UV measurements were performed with a Kinetex 2.6 μ Biphenyl 100 Å column (Phenomenex) equipped with a Phenylhexyl Security Guard ULTRA cartridge (Phenomenex). The mobile phases were 5 mM ammonium acetate with 0.5% v/v acetic acid in water, and acetonitrile (ACN) at a flow rate of 0.26 mL/min. A stepwise gradient was used: 25–55% ACN (5 min), 55–70% ACN (5–7.2 min), 70–90% ACN (7.2–7.5 min), 90% ACN was held (7.5–9 min), and then re-equilibrated to starting conditions (25% ACN). The compounds were detected at 254 nm (DAD).

For substrate and product quantification by GC, hexanoic acid, hexanal, and hexanol were measured in different concentrations (0.31–10 mM), respectively, to calculate standard curves for each compound. Concentrations were calculated using the linear interpolation. The samples were analyzed on an Agilent 6890N Network GC System equipped with a flame-ionization detector (FID), a Combi Pal injector (CTC Analytics, Zwingen, Switzerland) and a Chirasil-DEX CB column (25 m × 0.32 mm, 0.25 μm film, Agilent, Vienna, Austria). The following settings were used for the analyses: injector temperature: 200°C, detector temperature: 230°C, 2:1 split ratio, 2.0 mL/min constant N_2_ carrier gas flow; temperature programme: 40°C hold 5 min, 10°C/min to 140°C, 20°C/min to 160°C hold 7 min.

## Results

Multiple sequence alignments revealed a relatively small number of conserved residues (≤37, depending on the alignment program used) among the currently known four CAR subtypes, given the total number of CAR amino acids (~1,050–1,200) (Stolterfoht et al., 2017). The goal of this study was to identify essential residues for enzymatic carboxylate reduction within these conserved residues to broaden our understanding of carboxylate reductases (E.C. 1.2.1.30) in general, and type III CARs in particular. Our hypothesis was that the replacement of catalytically essential amino acids would result in inactive protein. Based on multiple sequence alignments of 26 recombinant CARs functionally characterized to date (Table 1), and homology models of *Nc*CAR generated to reveal putative active sites, *Nc*CAR variants with replacements of 34 highly conserved amino acids were generated. Specifically, 33 residues were replaced by alanine and one conserved alanine residue was replaced by a glycine. Subsequently, the *in vivo* and *in vitro* activity of these variants was explored, and residues playing a role in one of the catalytic substeps, or in substrate or cofactor binding identified. For the essential post-translational modification, PPTase from *E. coli* was co-expressed with *Nc*CAR from one plasmid. *E. coli* K12 MG1655 RARE served as host and the cells were cultivated as described previously (Schwendenwein et al., 2016).

### CAR expression and activity

Wild-type *Nc*CAR and each enzyme variant were purified by nickel-affinity chromatography and characterized by SDS-PAGE. Specific activities toward the reduction of cinnamic, piperonylic, and hexanoic acid were determined via a spectrophotometric NADPH depletion assay. Additionally, whole cell biotransformations were analyzed by HPLC-UV and GC-FID to detect the products and to assess the *in vivo* activity of *Nc*CAR variants compared to the wild-type CAR. The impact of replacements of conserved residues is summarized in Table 2.

#### Amino acid replacements that affect protein expression

The amino acid exchange G184A (Table 2, entry 1) in the A-domain and R870A (Table 2, entry 11) as well as W978A (Table 2, entry 12) in the R-domain was detrimental to soluble expression. The variants were found exclusively in the insoluble fraction (Supporting information, Figure S3). Similarly, the variant P904A (Table 2, entry 12) exhibited reduced soluble expression (Figures 1A, 2A).

#### Amino acid replacements that abolish activity

In the A-domain, the substitution of three amino acid residues (H237A, E337A, and E433A) abolished CAR activity as determined *in vitro* (Figure 1B). Notably, the variant E337A (Table 2, entry 4) retained some activity on piperonylic acid *in vivo* (Figure 1C). In the T-domain, no activity was found for enzyme variant S595A (Table 2, entry 7). Replacement of R-domain residues Y844 and K848 to alanine also led to activity loss (Table 2, entry 10, Figures 2B,C).

#### Amino acid replacements that reduce activity

A moderate decrease in activity was observed for the variants S183A and G457A, while the variants T336A, D405A, and R422A showed a stronger decrease in activity. Interestingly, the variant D405A (Table 2, entry 5) showed activity solely on piperonylic acid (*in vivo* and *in vitro*, Figures 1B,C). Similarly, the variant T336A (Table 2, entry 4) retained activity mainly on piperonylic acid (Figures 1B,C). The exchange of G310 for alanine (Table 2, entry 3) exhibited a specific activity with cinnamic acid similar to that of the wild-type, but a decreased activity with piperonylic and hexanoic acid. Only residual activity was observed for variants G691A and G694A (Table 2, entry 8). Furthermore, the activities of G882A and P904A were relatively low (Figures 2B,C).

#### Amino acid replacements that increase activity

**Figure 3:**
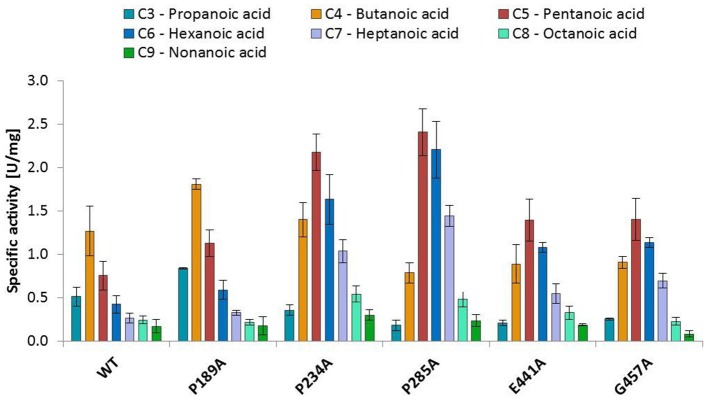
Activities of wild-type *Nc*CAR and selected variants for the reduction of aliphatic acids determined by following NADPH consumption at 340 nm for 10 min at 28°C. Data are shown as mean values of three separate experiments, carried out in four technical replicates, respectively. For the wild-type and the variants P234A, and P285A, data from biological duplicates were included.

The substitution of P285 with alanine seems to increase the activity for the reduction of piperonylic acid (Figure 1B). Furthermore, some substitutions with alanine (P189A, P234A, P285A, E441A, and G457A) enhanced the specific activity toward hexanoic acid compared to the wild-type. Therefore, we screened a spectrum of aliphatic acids from propanoic to nonanoic acid (Figure 3). Whereas wild-type *Nc*CAR showed the highest activity toward butanoic acid, for some variants the preference was shifted to pentanoic acid with an overall increased activity on aliphatic acids with longer chains, especially for variants P234A and P285A.

### Modeling of *Nc*CAR

**Figure 4:**
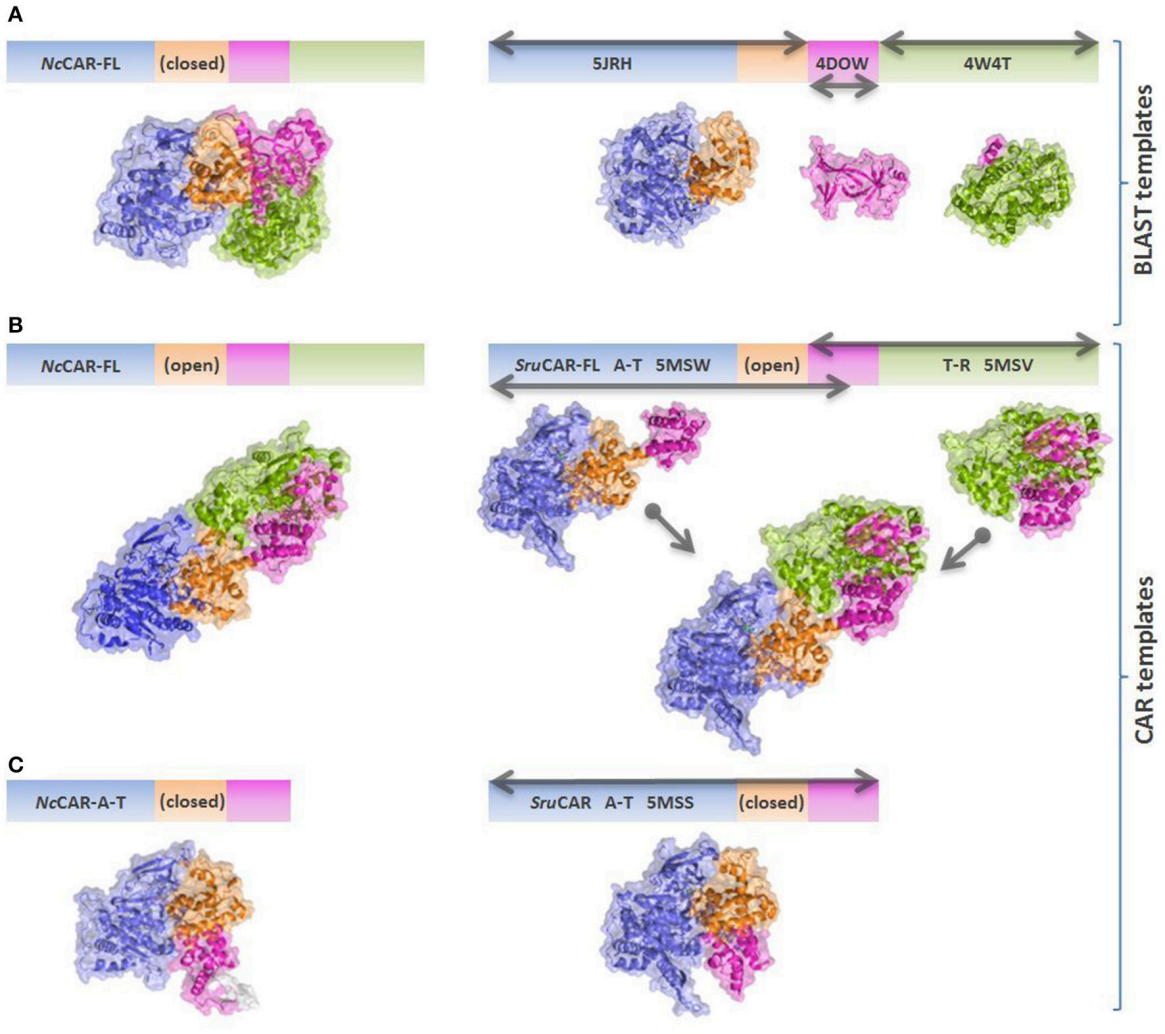
Schematic overview of *Nc*CAR models and templates. Models are colored according to individual domains as in Gahloth et al. (2017). Blue: A_core_; orange: A_sub_; magenta: T-domain; green: R-domain. Gray arrows indicate the parts of the sequence which are covered by the individual templates. **(A)** *Nc*CAR full length model based on best BLAST templates (mainly PDB codes 5JRH, 4DOW, and 4W4T). 5JRH: *Salmonella enterica* acetyl-CoA synthetase (Han et al., 2016); 4DOW: *Mus musculus* ORC1 BAH domain (Kuo et al., 2012); 4W4T: *Stigmatella aurantiaca* terminal reductase domain from the myxalamid NRPS MxaA (Barajas et al., 2015). **(B)** *Nc*CAR full length model based on the open *Sru*CAR structures, fused together to a full length *Sru*CAR template. **(C)** *Nc*CAR A-T-didomain model based on the closed *Sru*CAR A-T-didomain structure (PDB code 5MSS).

**Figure 5:**
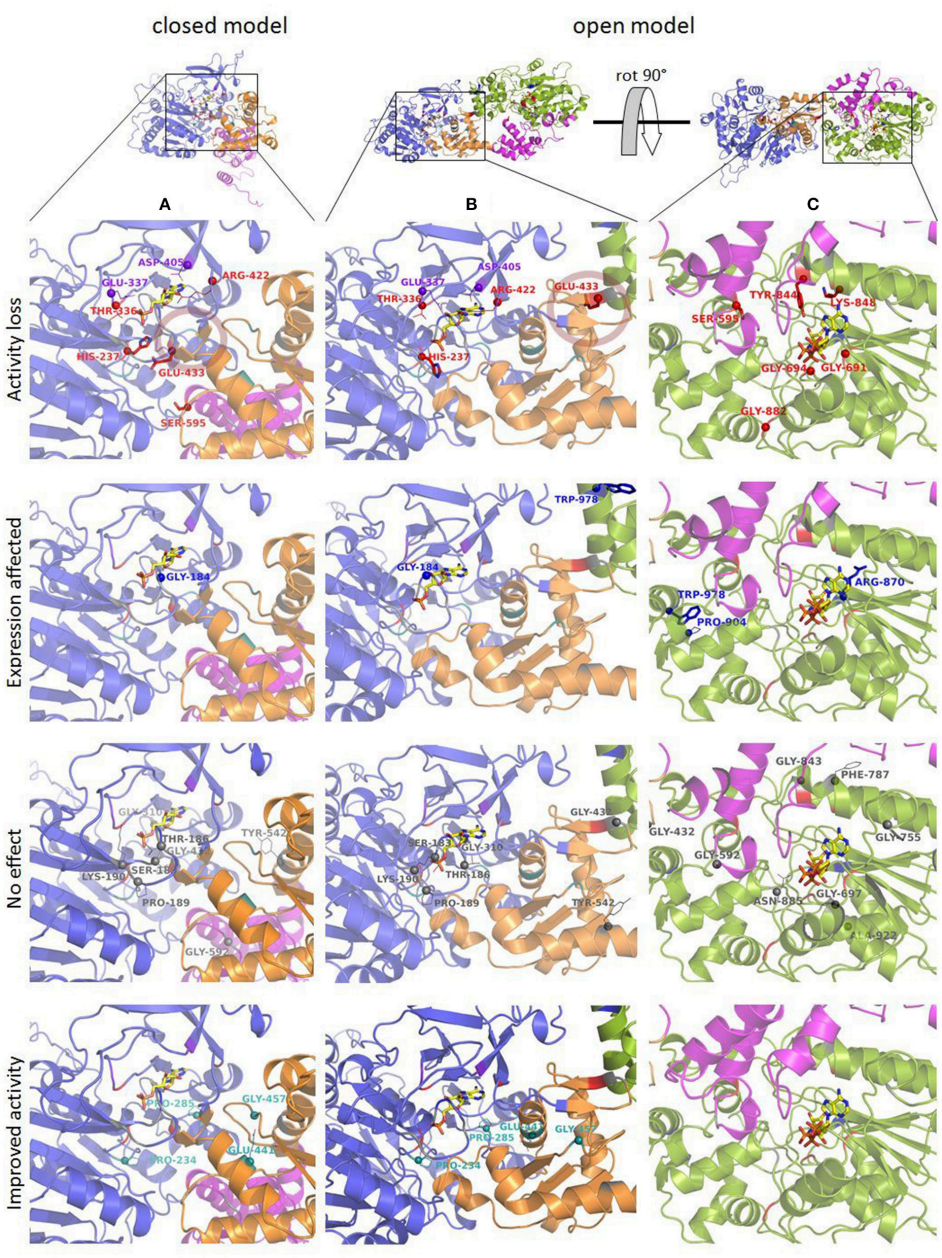
*Nc*CAR models based on *Sru*CAR templates. **(A)** *Nc*CAR model (closed) A-domain with selected amino acid exchange locations. Bound AMP is shown as yellow sticks; a transparent red circle indicates the location of two short beta sheets. **(B)** *Nc*CAR-FL model (open) A-domain with location of “shifted” Glu 433, located at the two short beta sheets. **(C)** Close-up of R-domain of *Nc*CAR-FL model with selected amino acid exchange locations. Bound NAP is shown as yellow sticks. Red spheres with amino acids as stick representation indicate selected residues which caused activity loss upon replacement by alanine. Amino acids with red line representation indicate variants where little activity was observed and purple spheres indicate locations where replacements with alanine retained activity solely on piperonylic acid. Blue spheres with amino acids as stick representation indicate amino acid exchange locations in the model where expression was abolished and amino acids indicated as blue lines where the expression was very low. Gray spheres indicate locations where, after substitution by alanine, activity was not significantly changed compared to the wild-type CAR. Teal spheres indicate amino acid exchanges where improved activity for hexanoic acid was observed.

An overview of the models created in this study is depicted in Figure 4. As the underlying sequence identities to the available templates are low, the resulting model needs to be analyzed with care. Figure 5 shows close-ups of the A-domain in a closed and open conformation and the R-domain with the key residues highlighted.

## Discussion

Aligning all experimentally confirmed CAR sequences with A-T-R architecture and broad substrate scope, a particular amino acid pattern becomes apparent that seems to be inherent to the carboxylate reductase function in all E.C. 1.20.1.30 CARs. To gain some insight into the overall structure arrangement and possible locations of the binding pockets, we created *Nc*CAR models (Figures 4, 5). We used this information to predict cofactor and substrate binding sites (Table 2, suggested function). We hypothesized that some of the conserved residues would be important key residues for catalysis and therefore performed an alanine scan, assuming that replacement of a key residue by alanine would abolish enzymatic carboxylate reduction activity.

### Key residues in the A-domain

We assume His 237 of the Pxx**H** motif (Table 2, entry 2), and Glu 433 of the L(F)S(A)xG**E**K(F) motif (Table 2, entry 5) are essential residues of the A-domain, because their replacement results in complete activity loss. According to the *Nc*CAR model, His 237 may interact with a potential substrate and the phosphate of AMP (Figures 5A,B). His 237 (numbering according to *Nc*CAR, His 300 in *Ni*CAR) was previously recognized to be conserved in CAR enzymes and to be located close to both AMP phosphate and the substrate carboxylate (Gahloth et al., 2017). To the best of our knowledge, the essential Glu 433 was studied experimentally herein for the first time. Glu 433 was interesting as it is positioned at the hinge region (two short beta strands, transparent red circles, Figures 5A,B), which is pointing in opposite directions in the closed and open models. In the closed model, it is near to His 237 and in the open model it is distant from the binding pockets. As explained by Gahloth et al. the open form created by rotation of the A-T-didomain, may not be as pronounced in solution and is maybe stabilized by crystal contacts upon the crystallization process. The distance of Glu (E533 in *Sru*CAR) to the essential His (H315 in *Sru*CAR) is also indicated in Figures 2c,d in Gahloth et al. (2017). Our model confirms this observation (Figures 5A,B).

### Role of other conserved residues of the A-domain

Like His 237, Ser 183 and Thr/Ser 182 (Table 2, entry 1) and Thr 336 (entry 4) may interact with the AMP cofactor according to our *Nc*CAR model. The S(T)**SG**S(T)**T**G(S)x**PK** motif (Table 2, entry 1) resembles the highly conserved core motif A3 of NRPS defined by Marahiel et al. (1997), to which an interaction with the pyrophosphate leaving group was assigned (Conti et al., 1997). However, the substitution of the Ser, Thr, Pro, or Lys of this motif in CARs did not significantly impact on CAR activity. Other core motifs of NRPS defined by Marahiel et al. share also similarity with signature sequences of CARs defined in Table 2. For example, the core motif A5 is akin to the G(S)**TE**xG(T) motif (Table 2, entry 4). Thr 336 and Glu 337 of this motif (Table 2, entry 4) as well as Asp 405 and Arg 422 of the **D**x_14−17_**R** motif (entry 5) are not considered essential, because their exchange for alanine resulted in active variants (Figures 1B,C). Asp 405 and Arg 422 may be involved in interactions with the adenosine ribose according to our model (Figures 5A,B).

### Key residue of the T-domain

As already reported by others, the replacement of the phosphopantetheine binding site Ser 689 in *Ni*CAR by Ala resulted in an inactive variant (Venkitasubramanian et al., 2007; Wood et al., 2017). The same is true for the equivalent exchange of Ser 595 in *Nc*CAR [Table 2, entry 7; or core motif T (core 6) in Marahiel et al. (1997)], confirming that this residue serves as the phosphopantetheine attachment site and is indispensable for CAR activity.

### Key residue of the R-domain

In the R-domain, Tyr 844, and Lys 848 of the G**Y**xxx**K**xxxE(S) motif (Table 2, entry 10) are essential for catalysis, as their substitution with alanine led to complete inactivation of *Nc*CAR. Wang and co-workers revealed that the R-domain of *At*CAR shares the conserved patterns of TGxxGxxG and YxxxK with short-chain dehydrogenase/reductase (SDR) family proteins (Wang et al., 2014). Previous studies on SDRs assigned a structural role in the coenzyme binding region to the TGxxGxxG motif, and suggested YxxxK to be part of the active site in SDR proteins (Persson et al., 2003). Several site-directed mutagenesis studies on SDR enzymes involved the conserved residues of these motifs and confirmed their importance as their substitution abolished enzyme activity (Chen et al., 1990; Albalat et al., 1992; Obeid and White, 1992; Cols et al., 1993; Chhabra et al., 2012; Barajas et al., 2015). For CARs, a first comparable study to analyse the contribution of these conserved residues to catalytic activity was carried out by Wang and co-workers. Therefore, T690 of the TGxxGxxG motif, as well as the active site Y863 and K867 of *At*CAR were mutated. The activities of the T690A and Y863F variants on 5-methyl orsellinic acid (5-MOA) were drastically reduced, whereas the exchange of Lys 867 for Ala led to a completely inactive enzyme (Wang et al., 2014), which agrees with our study of the corresponding residues in *Nc*CAR (Y844 and K848).

### Role of other conserved residues of the R-domain

The first two glycines of the T(S)**G**xx**G**xxG motif (Table 2, entry 8) are important residues of the R-domain, because their replacement resulted in drastic reduction of activity (Figures 2B,C). This motif is well known as “Rossmann-fold” dinucleotide cofactor binding motif. In the *Nc*CAR model, the R-domain (Figure 5C) clearly shows an “extended” type of NADPH-binding α/β Rossmann fold, which is composed of seven parallel beta strands in a twisted beta sheet arranged in the order 3-2-1-4-5-6-7. The signature sequence T/S**G**xx**G**xxG at the region of the tight turn at the end of the first β-strand was reported to be in contact with the negatively charged oxygens of the two phosphates of NADPH. The first glycine was proposed to be important for the tightness of the turn, the second allows the dinucleotide to be bound without obstruction from an amino acid side chain at this position, and the third seems to provide space for a close interaction between the β-strand and the succeeding α-helix. Other conserved features of this fingerprint region are (i) a hydrophilic residue at the N-terminus of the first β-strand, (ii) six small hydrophobic residues that form a hydrophobic core of the βαβ unit, and (iii) in case of NADP(H) binding: a basic residue in the loop after the second β-strand, as observed in the extended type of SDRs (Wierenga et al., 1985; Scrutton et al., 1990; Lesk, 1995). Except for the first of the three above-mentioned conserved features, all these characteristics are in accordance with our *Nc*CAR model. The basic residue mentioned above is probably a highly conserved Arg, located about 20 residues downstream of the third Gly (R718 in *Nc*CAR; Table 2, entry 8).

The comparison of the presumable NADPH binding site of our model with available protein structures of members of the SDR family provided an indication for other residues involved in the reduction mechanism or in NADPH binding. For example Breton et al. reported that a conserved aspartic acid residue, found in the loop between β3 of the seven-stranded β-sheet and α3, interacts with the adenine moiety of NADP^+^ (Breton et al., 1996). In *Nc*CAR we also found an Asp in this loop. It seems to be conserved in CARs, except for *Tv*CAR, which has a valine at this position. Based on our *Nc*CAR models, the side chain of Asp 748 could maybe interact with the adenine.

Consistent with previous structural studies on the SDR superfamily, the nicotinamide moiety of the cofactor is pointing toward the active site (Figure 5C). Besides the YxxxK motif (Table 2, entry 10), a Ser or Thr constitutes the catalytic triad (Jörnvall et al., 1995; Oppermann et al., 1997), which is most likely located at position 815 in *Nc*CAR. At this position, there is a Ser exclusively in fungal CARs, whereas in bacterial CARs, Thr is strictly conserved (Table 2, entry 10). In most SDRs an essential Asn residue (or another Ser), that could be connected through a water molecule to the active site Lys, is extending this triad to form a catalytic tetrad (Filling et al., 2002). Such a residue could not be found in our models or is absent in CARs. According to the concept of the reductive reaction mechanism postulated for SDRs, the Tyr donates a proton to the oxygen of the substrate carbonyl, Ser stabilizes the deprotonated state of Tyr, and the pro-S hydride of the nicotinamide nucleophilically attacks the carbon of the substrate carbonyl. A proton relay is formed that involves the 2′OH of the nicotinamide ribose, which is stabilized by the Lys side chain, and a water molecule bound to the backbone carbonyl of the Asn (Breton et al., 1996; Filling et al., 2002). The presence of at least two, if not all, residues of the catalytic triad/tetrad in CARs lets us assume a similar reduction mechanism. The major role of the Lys was proposed to be the stabilization of the dinucleotide through the interaction with the nicotinamide ribose. In the model discussed herein, the ε-amino group of Lys 848 is pointing away from the 2′-hydroxyl of the nicotinamide ribose.

Finally, replacement of Gly 882 of the **G**xx**N**xxD/E motif (Table 2, entry 1) by Ala led to significantly decreased activity (Figure 2B). Asn 885 may interact with the pyrophosphate of the dinucleotide and is on the open side of the cavity, therefore also accessible to potential substrate interactions. Another speculation on the basis of the *Nc*CAR model is that the acidic residue of this motif interacts with the substrate.

### Substrate specificity conferring residues

Variant P234 reduced piperonylic acid less well compared to the wild-type, but showed increased activity for hexanoic acid. Variant P285A was outstanding for both hexanoic acid and piperonylic acid (Figure 1B). We propose that specific residues impart certain specificities, which would allow engineering of CARs for an increased activity toward a desired substrate. To test this hypothesis, we determined specific activities of five alanine variants for the reduction of a range of aliphatic acids and indeed saw distinct substrate preference patterns. Replacement of the two prolines P234 and P285 with alanine elevated the activity for longer aliphatic acids (Figure 3). Since both prolines are possibly located at the margin of the binding pocket – Pro 234 is in proximity of the essential His 237 residue, and Pro 285 is located in a turn between a short β-sheet and an α-helix – without the rigidity or flexion of the peptide chain conferred by proline, both replacements may change the cavity in a way favorable for binding longer fatty acids. In addition, a specificity shift toward longer aliphatic acids was observed for the variants E441A and G457A (Figure 3).

When Gross et al. isolated the *Nc*CAR enzyme from the fungus SY7A and characterized its substrate specificity, no activity with aliphatic acids, and in particular with hexanoic acid (caproic acid) was detected (Gross and Zenk, 1969; Gross, 1972). A possible explanation for the discrepancy between our and Gross result regarding substrate scope of *Nc*CAR could be that we deal with different enzyme variants in *N. crassa* strain SY7A and OR74A, respectively, or the recombinantly expressed enzyme lacks so far undescribed post-translational modifications.

Our findings support the proposed “gate-keeper”/substrate recognition function of the A-domain (Wang and Zhao, 2014; Moura et al., 2016; Finnigan et al., 2017), as the mutational exchanges in the A-domain led to either increase or decrease in activity while no activity enhancements were observed when residues of the R-domain were replaced.

### Amino acid substitutions that affect CAR expression

The replacement of Gly 184, Arg 870, and Trp 978 by alanine did not result in soluble expression. Pro 904 is located close to Trp 978, and its substitution by Ala yields significantly less soluble protein (Figure 2A). The *in vitro* activity of this variant was very low (Figure 2B), however, the *in vivo* activity of P904A was comparable to the wild-type activity (Figure 2C). This was also observed for variant G882A, and some other variants, especially for E337A, which showed virtually no activity *in vitro*, but clearly exhibited activity on piperonylic acid in the whole cell biotransformation (Figures 1B,C). A reason therefore could be protein stability.

### Outlook on probing further residues

We restricted this mutagenesis study to strictly conserved amino acids. Furthermore, only variants with single amino acid exchanges and substitutions only for alanine (or in case of alanine, for glycine) were generated. However, it is to be expected that prospective mutagenesis studies could broaden our understanding of the function of each residue present in signature sequences and could even reveal further essential residues or residues that impart specificities for certain substrates. Additional residues that are suitable for exchange studies are those not strictly conserved, but conserved regarding their biochemical property (e.g., acidic, aromatic, hydroxylic, aliphatic). For example, the threonine in the motif of three glycines (Table 2, entry 8), that is characteristic of NAD(P)(H)-binding enzymes, was not replaced, as it is not completely conserved, albeit only in *Ms*CAR2 and *Ms*CAR3 a serine with similar biochemical property occupy this position. The three aliphatic residues upstream of this threonine are likewise conserved. Considering amino acids possessing comparable biochemical property, many more positions than the 34 which were examined in this study are conserved, e.g., the hydroxylic residues neighboring on Ser 183 and Gly 184 (Table 2, entry 1), an acidic amino acid four residues downstream of the conserved Pro 285 (entry 3), or an aromatic amino acid upstream of the conserved Tyr 542 (Table 2, entry 7), to name just a few. Other positions are often only conserved among bacterial CARs. For example while bacterial CARs share an aromatic Phe after the conserved Gly 432 and Glu 433 in entry 6, there is a basic lysine at the respective position in fungal CARs (*Nc*CAR, *Tv*CAR, *At*CAR, and *Sb*CAR). Another example is the universally conserved Lys from ANL motif A10 (Marahiel et al., 1997), involved in polar interactions with both the adenosine and the nucleotide ligand, highly conserved in bacterial CARs (Gahloth et al., 2017), but not in fungal CARs, where there is a hydroxylic residue in place of this Lys (Thr 524 in *Nc*CAR, not listed in Table 2). As Wang and Zhao engineered *At*CAR toward their target substrate anthranilate they also found a hydroxylic residue (Ser 540 in *At*CAR) occupying the same position as the lysine previously reported to be absolutely conserved in other A-domains of NRPSs. Their homology model suggested that the distinct Ser, other than Lys at this position, is not involved in any interaction with the ligand (Wang and Zhao, 2014). However, there is a Lys two amino acids upstream in fungal CARs (Lys 522 in *Nc*CAR) which could provide these key interactions as it points toward the adenosine ribose in the open model but is directed outward in the closed model. Another position to be mentioned is the conserved key Asp residue (D984 in *Ni*CAR), of which two distinct conformations in the reductase active site were observed [the orientation of this residue was postulated to lead to an on/off state of the reductase (Gahloth et al., 2017)], which is occupied by a Gly in fungal CARs (G872 in *Nc*CAR, Table 2, entry 11). Since all characterized CARs show relaxed substrate scope, and no significant preferences for a particular substrate class can be assigned to one phylogenetic subgroup of CARs so far, no specificity conferring code can be deduced from these positions.

## Conclusion

To expand the knowledge about the structure and molecular functionality of CARs, the identification of key residues for the enzymatic reaction is of importance. The function of conserved residues was studied by a multiple sequence alignment and mutational replacements of these residues in a fungal CAR from *N. crassa*. The effect of these replacements was investigated by analyzing enzymatic activity *in vivo* and *in vitro*. The interpretation of the impact of the amino acid exchanges was supported by homology modeling and comparisons to related structures.

Residues H237, E433, S595, Y844, and K848 were identified to be essential for carboxylate reductase function. Residues T336, E337, D405, R422, G691, G694, and G882 seem to be also important for activity since the exchange to Ala significantly reduced CAR activity. Replacement of P234, P285, E441, and G457 by alanine increased the activity for hexanoic acid reduction, therefore, we assume that these residues play a key role in substrate binding. Our attempts to model *Nc*CAR gave a model of rather limited quality at this point, because identities to structural templates were generally very low. Nevertheless, the model allowed us to speculate on the function of several conserved residues.

*N. crassa* CAR readily reduced all tested aliphatic acids from C3 to C9. The wild type protein showed its highest activity for butanoic acid, but substitution of P234 and P285 resulted in a shift of the substrate preference toward pentanoic acid, and a higher specific activity for aliphatic acids compared to the wild-type *Nc*CAR in general. This study is a first comprehensive investigation of conserved residues by means of mutagenesis, and the herewith identified signature sequences of E.C. 1.2.1.30 CARs may help in the identification of new proteins with CAR activity.

## Author contributions

MW led the project, conceived the research and designed wet lab experiments. GS and KG designed the modeling. HS prepared and purified mutants, carried out in vitro and in vivo assays, analyzed the results and designed figures. DS prepared mutants and developed the protocol for growth in MTPs. GS and TP-K conducted the modeling. HS, GS, and MW co-wrote the manuscript. All authors commented on the manuscript.

### Conflict of interest statement

The authors declare that the research was conducted in the absence of any commercial or financial relationships that could be construed as a potential conflict of interest.
