# Supplementary material for: Identification of Key Residues for Enzymatic Carboxylate Reduction
Source: Front Microbiol. 2018 Feb 19;9:250. doi: 10.3389/fmicb.2018.00250 (PMC5826065; doi:10.3389/fmicb.2018.00250)
Supplement: Supplementary file 1 [file Table1.DOCX]

**Supporting information**

**Identification of key residues for enzymatic carboxylate reduction**

Holly Stolterfoht,^1,2^ Georg Steinkellner,^2,3^ Daniel Schwendenwein,^2^ Tea Pavkov-Keller,^2,3^ Karl Gruber,^2,3^ Margit Winkler^1,2^

^1^ Institute of Molecular Biotechnology, TU Graz, Petersgasse 14, 8010 Graz, Austria

^2^ Austrian Centre of Industrial Biotechnology, Petersgasse 14, 8010 Graz, Austria

^3^ Institute of Molecular Biosciences, University of Graz, Humboldtstraße 50, 8010 Graz, Austria

**SI content**

[Figure S 1. SDS-PAGE of Ni-affinity chromatography purified *Nc*CAR wild-type and variant enzymes. 2](#_Toc503054176)

[Figure S 2. SDS-PAGE of cell-free extracts (CFEs) of *Nc*CAR wild-type and variant enzymes. 3](#_Toc503054177)

[Figure S 3. SDS-PAGE of insoluble fractions (IFs) of *Nc*CAR wild-type and variant enzymes. 4](#_Toc503054178)

[Table S 1. Primers used for site-directed mutagenesis. 5](#_Toc503054179)

**A_sub_**

**A_core_**

**A**

**G184A**

**[kDa]**

**140**

**115**

**80**

**65**

**50**

**40**

**30**

**25**

**15**

**10**

**WT**

**S183A**

**G184A**

**T186A**

**P189A**

**K190A**

**P234A**

**H237A**

**P285A**

**G310A**

**T336A**

**E337A**

**D405A**

**R422A**

**G432A**

**E433A**

**E441A**

**G457A**


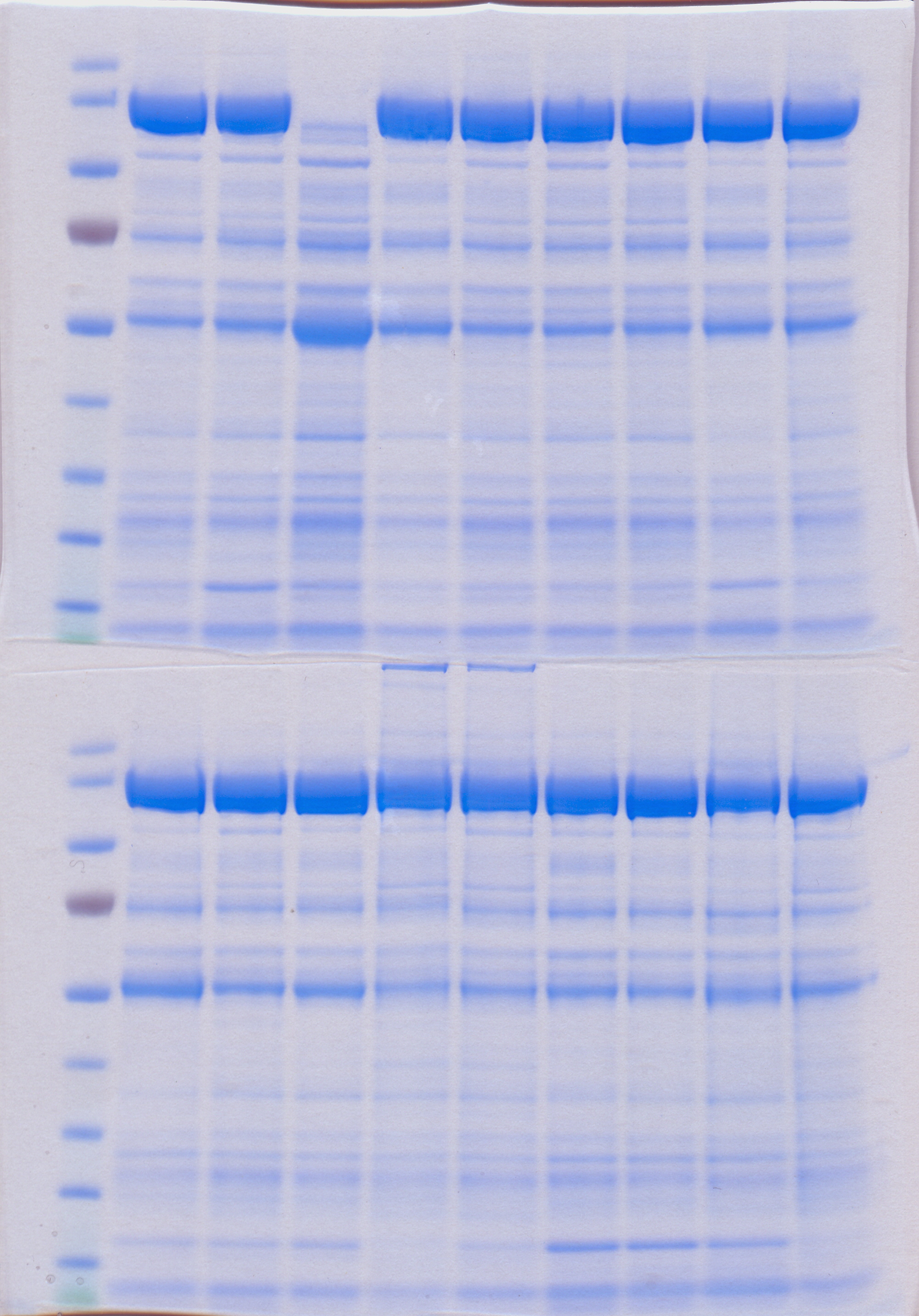

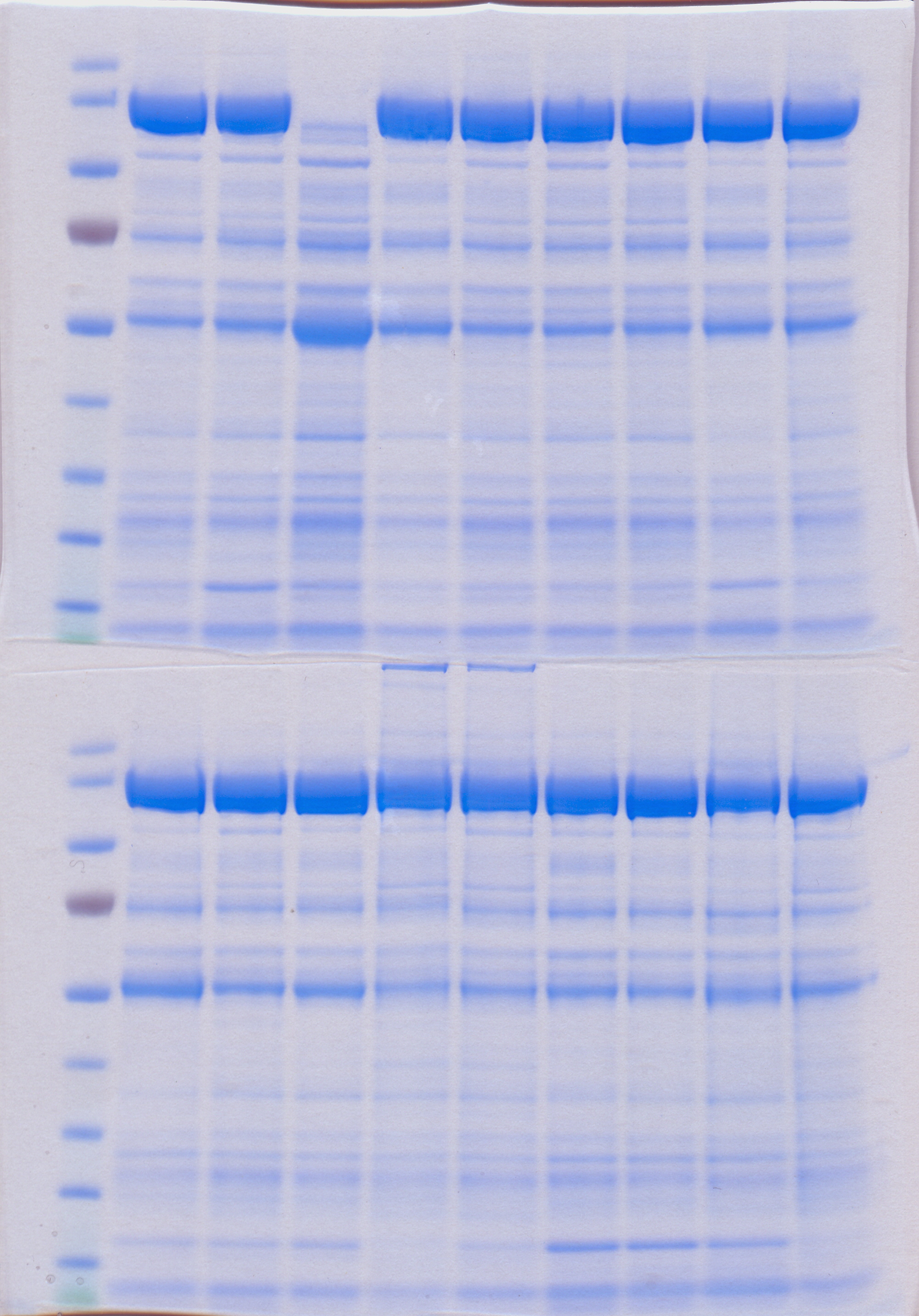


**R**

**T**

**A_sub_**

**B**

**W978A**

**P904A**

**R870A**

**[kDa]**

**140**

**115**

**80**

**65**

**50**

**40**

**30**

**25**

**15**

**10**

**WT**

**Y542A**

**G592A**

**S595A**

**G691A**

**G694A**

**G697A**

**G755A**

**F787A**

**G843A**

**Y844A**

**K848A**

**R870A**

**G882A**

**N885A**

**P904A**

**A922G**

**W978A**


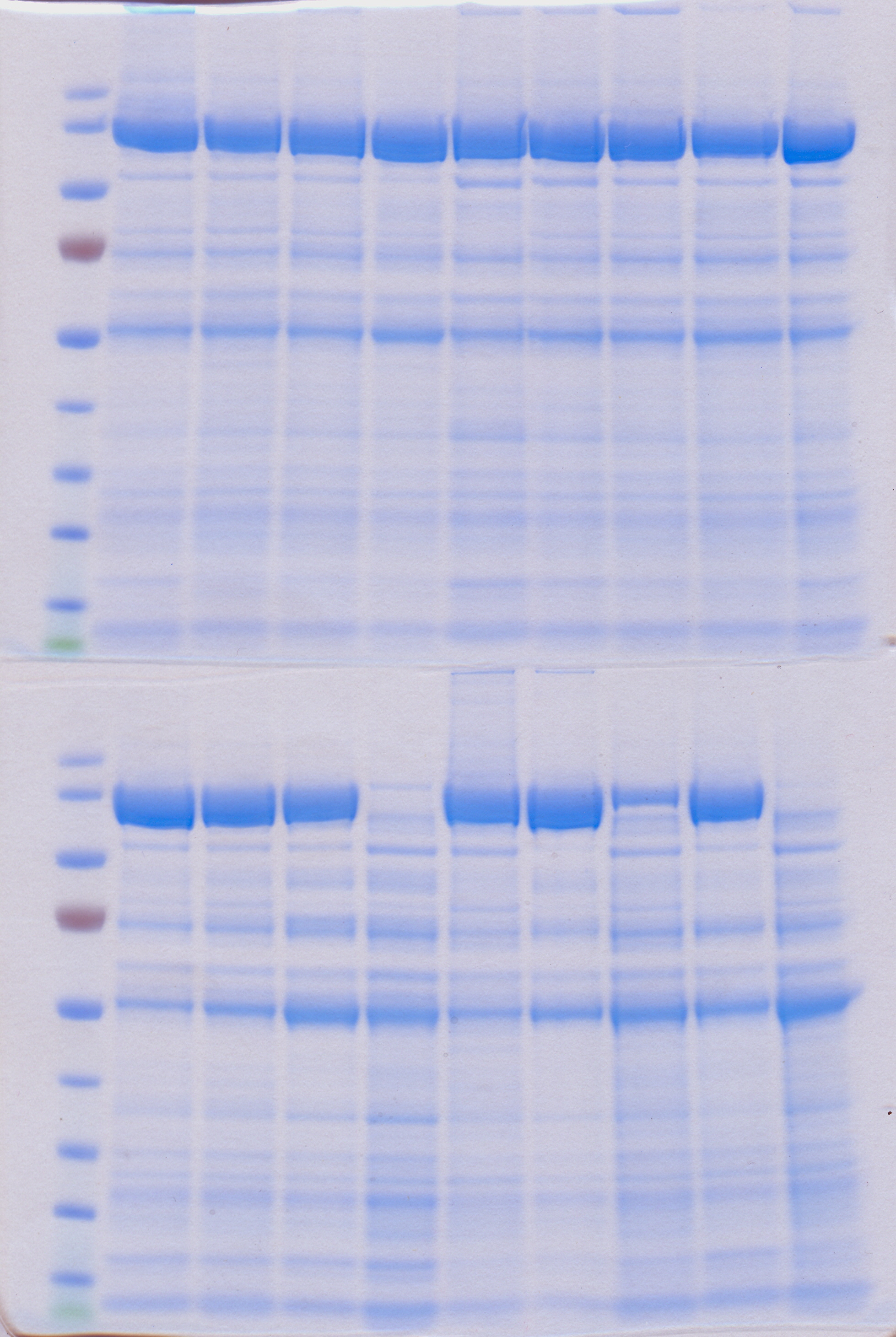

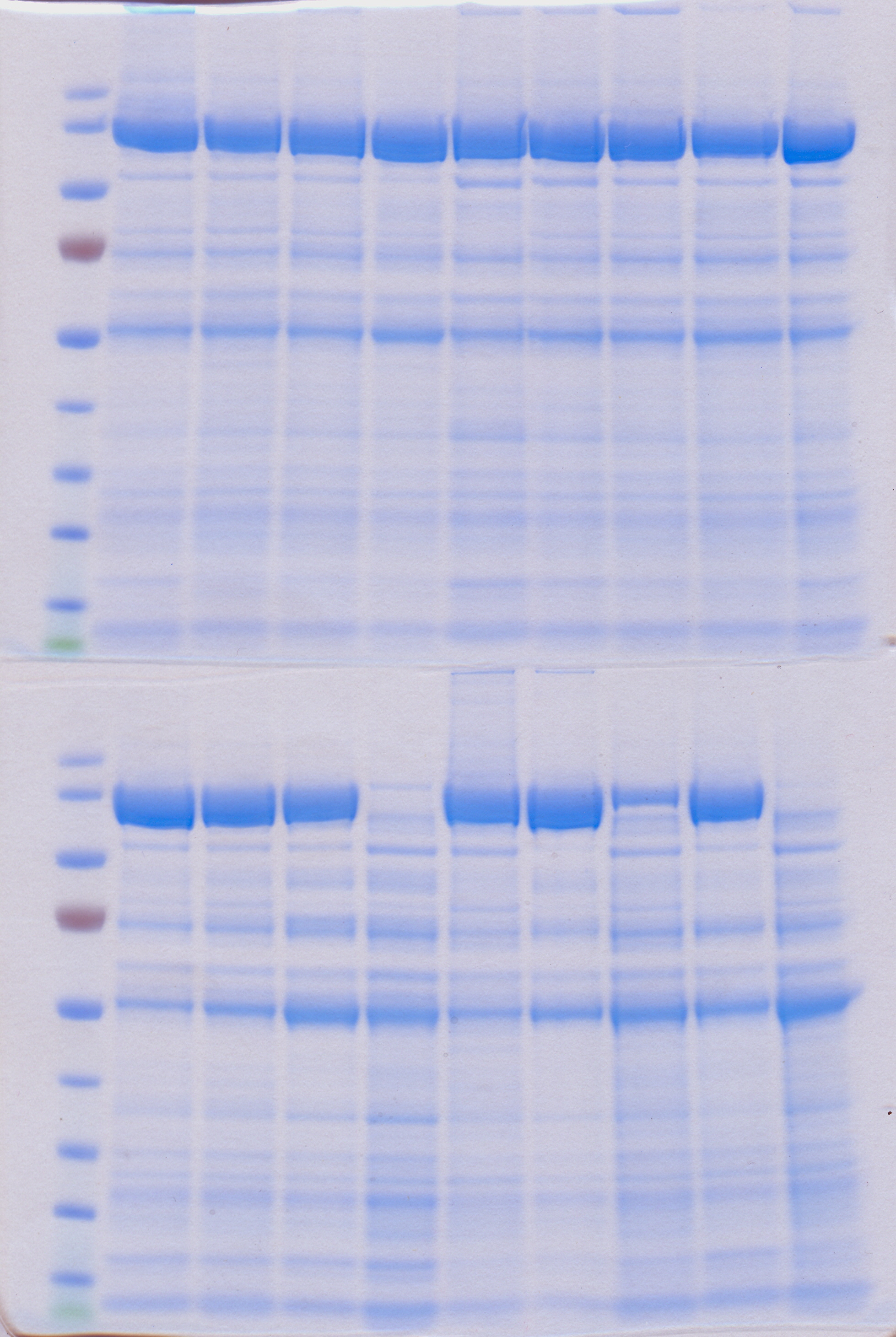


Figure S 1. SDS-PAGE of Ni-affinity chromatography purified *Nc*CAR wild-type and variant enzymes.

(**A**) Conserved amino acid residues located in the A_core_- and A_sub_-domain exchanged for alanine. Location is indicated by a blue (A_core_) or orange (A_sub_) bar. (**B**) Conserved residues located in the A_sub_-, T-, and R-domain substituted with alanine. Location is indicated by an orange (A_sub_), magenta (T), or green (R) bar. 10 µg of protein were loaded, respectively. The expected molecular weight of *Nc*CAR is 120 kDa (Schwendenwein et al., 2016). No soluble expression is visible for variant G184A, R870A, and W978A (highlighted in blue and underlined). Variant P904A showed lower CAR expression than WT (highlighted in pale blue). Ladder: PageRuler^TM^ Prestained Protein Ladder (Thermo Scientific). NuPAGE^TM^ 4-12% Bis-Tris gels (Thermo Scientific) were run for 50 min at 200 V and 120 mA in MOPS buffer and stained with SimplyBlue^TM^ SafeStain (Invitrogen).

**A_sub_**

**A_core_**

**A**

**[kDa]**

**140**

**115**

**80**

**65**

**50**

**40**

**30**

**25**

**15**

**10**

**G184A**

**WT**

**S183A**

**G184A**

**T186A**

**P189A**

**K190A**

**P234A**

**H237A**

**P285A**

**G310A**

**T336A**

**E337A**

**D405A**

**R422A**

**G432A**

**E433A**

**E441A**

**G457A**


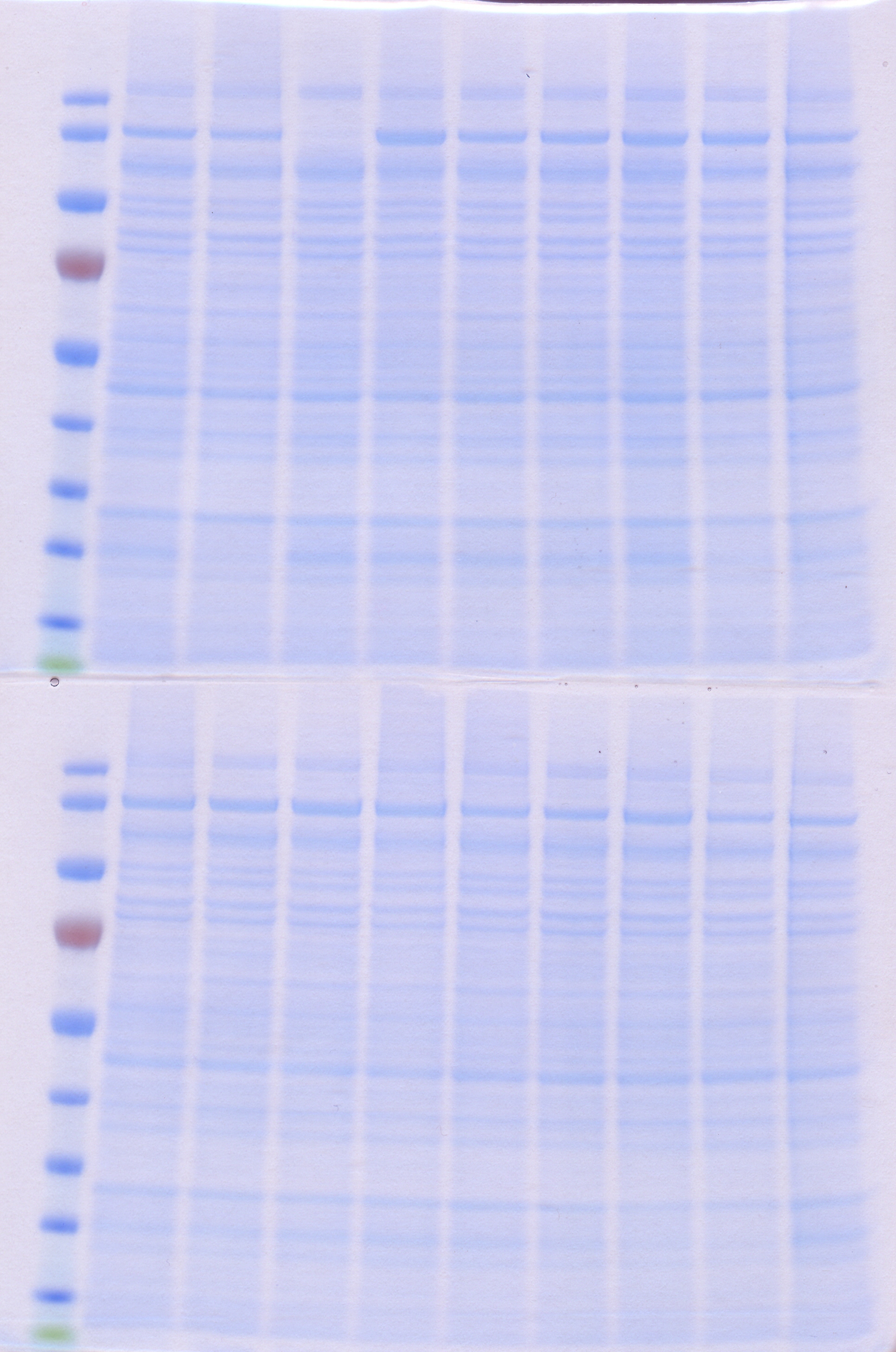

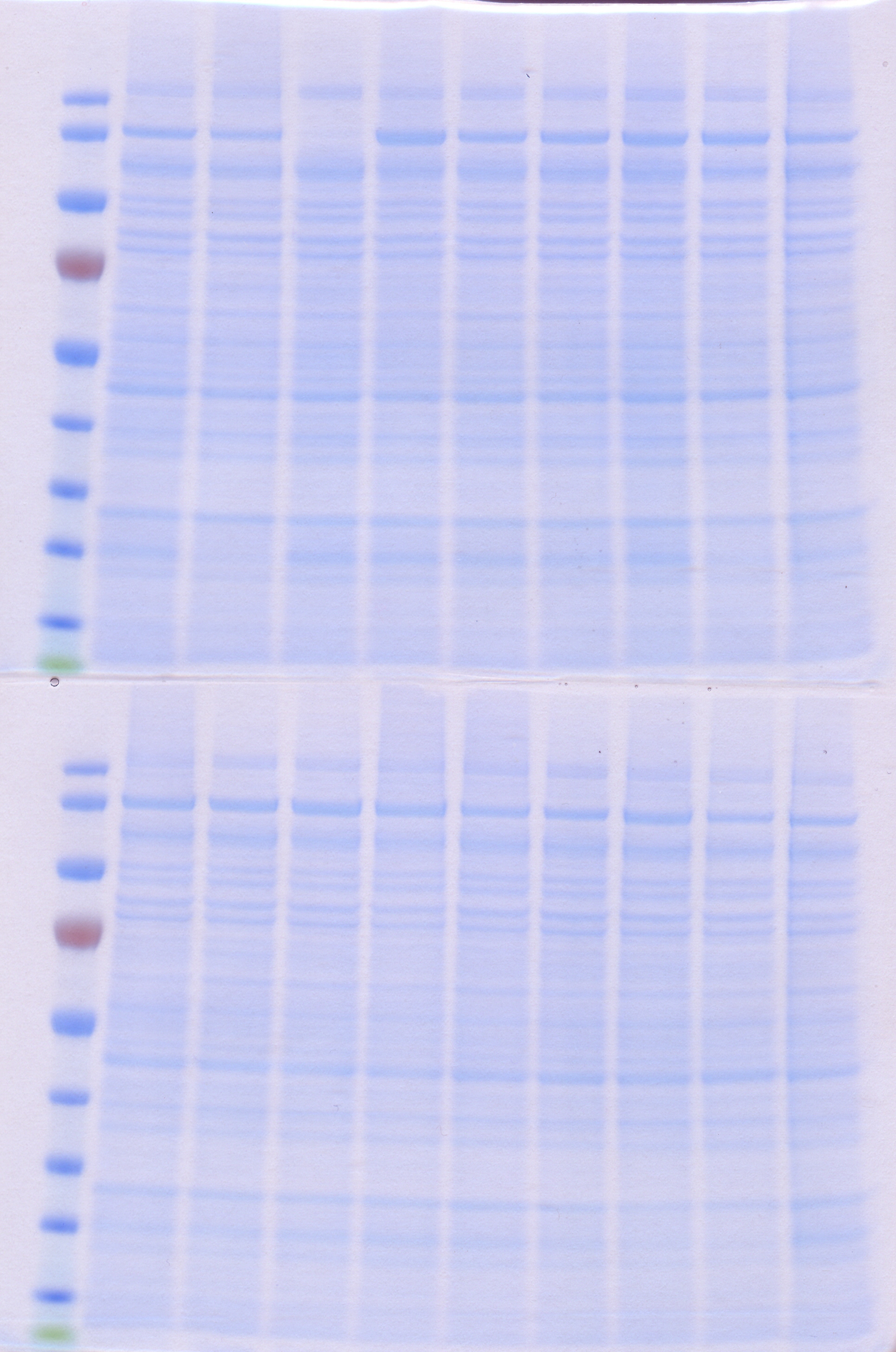


**A_sub_**

**T**

**R**

**B**

**P904A**

**R870A**

**W978A**

**[kDa]**

**140**

**115**

**80**

**65**

**50**

**40**

**30**

**25**

**15**

**10**

**Y542A**

**G592A**

**S595A**

**G691A**

**G694A**

**G697A**

**G755A**

**F787A**

**G843A**

**Y844A**

**K848A**

**R870A**

**G882A**

**N885A**

**P904A**

**A922G**

**W978A**

**WT**


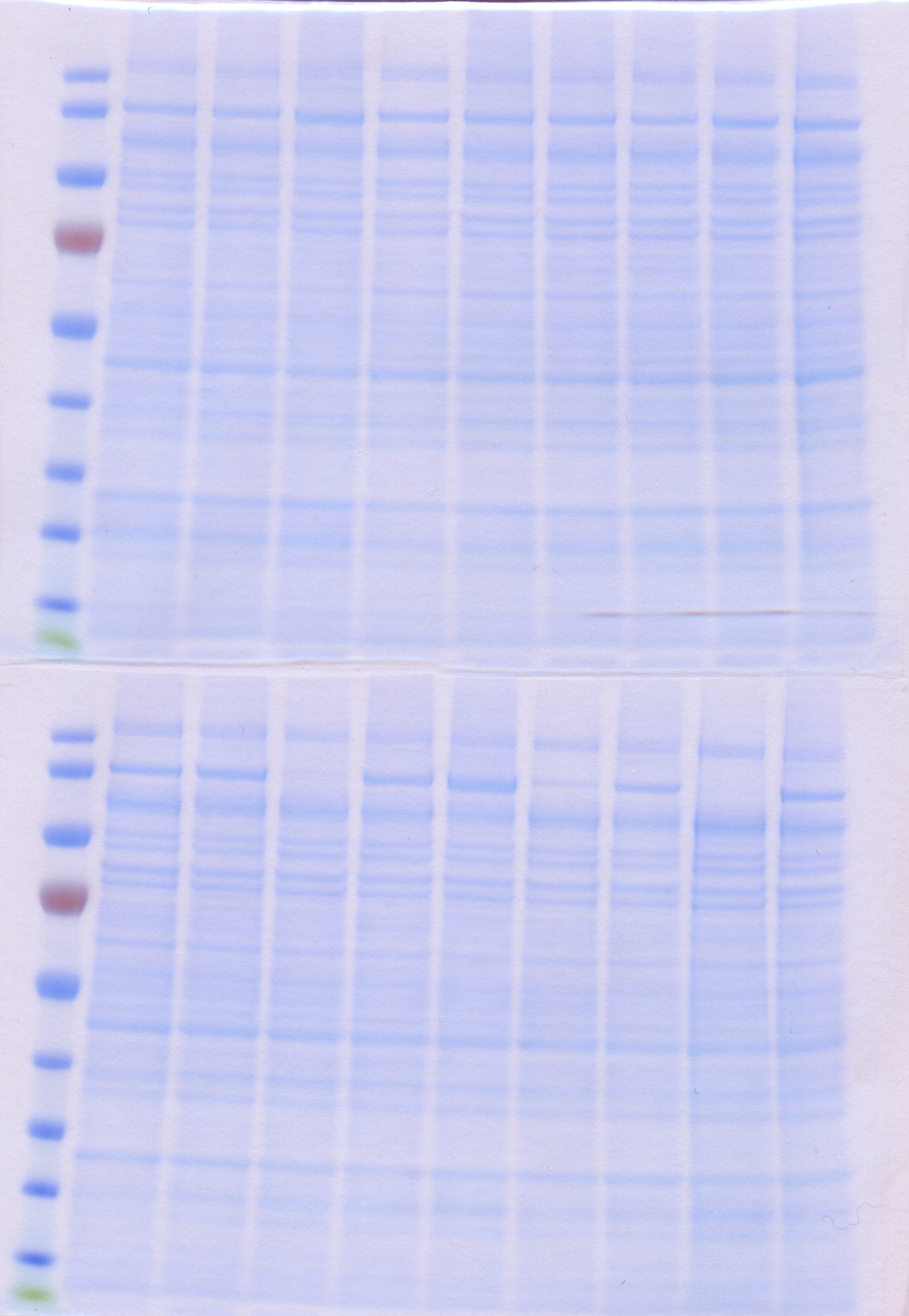

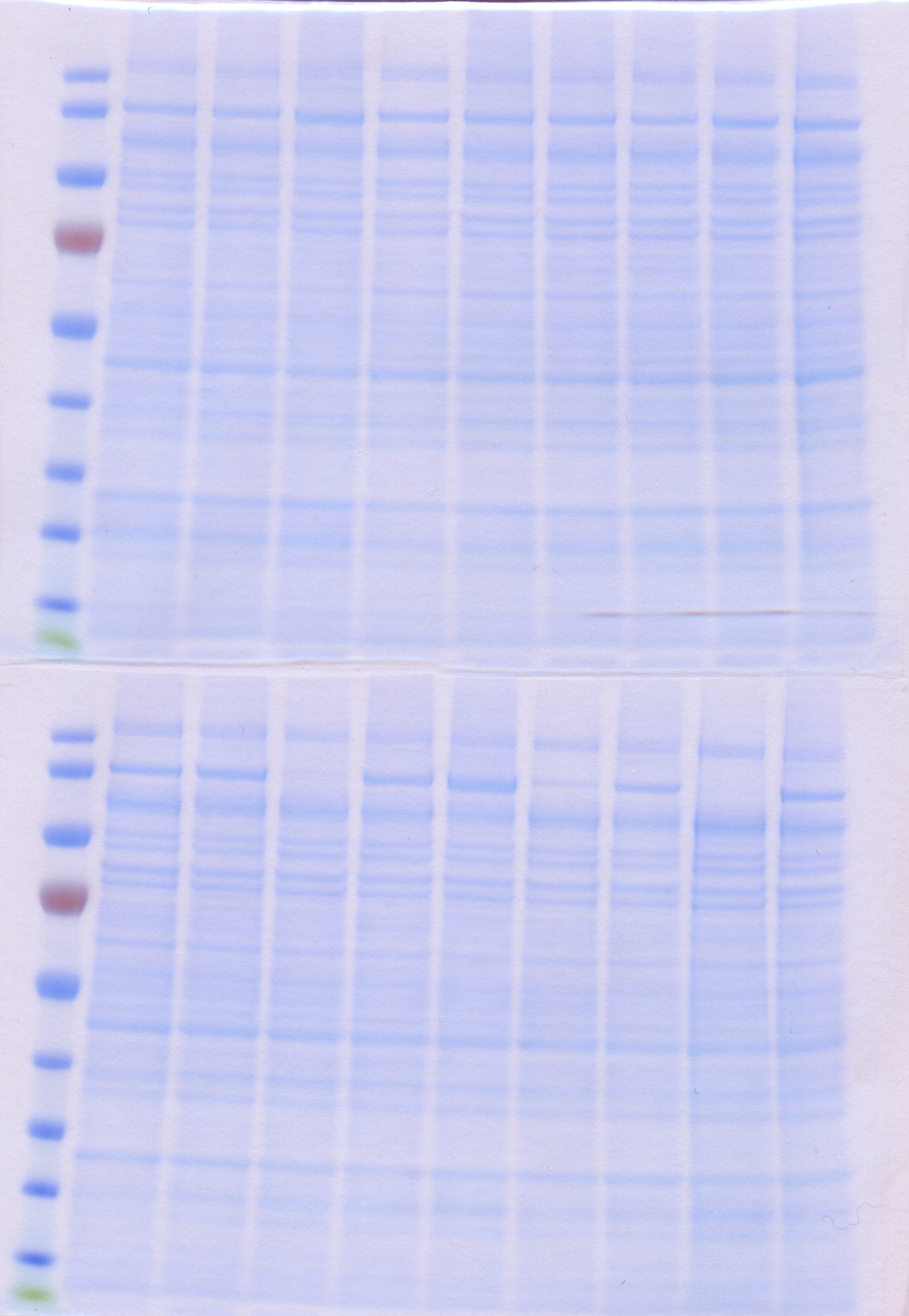


Figure S 2. SDS-PAGE of cell-free extracts (CFEs) of *Nc*CAR wild-type and variant enzymes.

CFEs were obtained after cell disruption by sonication and centrifugation for 1 h at 20,000 x g and 4°C. (**A**) Conserved amino acid residues located in the A_core_- and A_sub_-domain exchanged for alanine. Location is indicated by a blue (A_core_) or orange (A_sub_) bar. (**B**) Conserved residues located in the A_sub_-, T-, and R-domain substituted with alanine. Location is indicated by an orange (A_sub_), magenta (T), or green (R) bar. 10 µg of protein were loaded, respectively. The expected molecular weight of *Nc*CAR is 120 kDa (Schwendenwein et al., 2016). No soluble expression is visible for variant G184A, R870A, and W978A (highlighted in blue and underlined). Variant P904A showed lower CAR expression than WT (highlighted in pale blue). Ladder: PageRuler^TM^ Prestained Protein Ladder (Thermo Scientific). NuPAGE^TM^ 4-12% Bis-Tris gels (Thermo Scientific) were run for 50 min at 200 V and 120 mA in MOPS buffer and stained with SimplyBlue^TM^ SafeStain (Invitrogen).

**A_sub_**

**A_core_**

**A**

**[kDa]**

**140**

**115**

**80**

**65**

**50**

**40**

**30**

**25**

**15**

**10**

**G184A**

**WT**

**S183A**

**G184A**

**T186A**

**P189A**

**K190A**

**P234A**

**H237A**

**P285A**

**G310A**

**T336A**

**E337A**

**D405A**

**R422A**

**G432A**

**E433A**

**E441A**

**G457A**


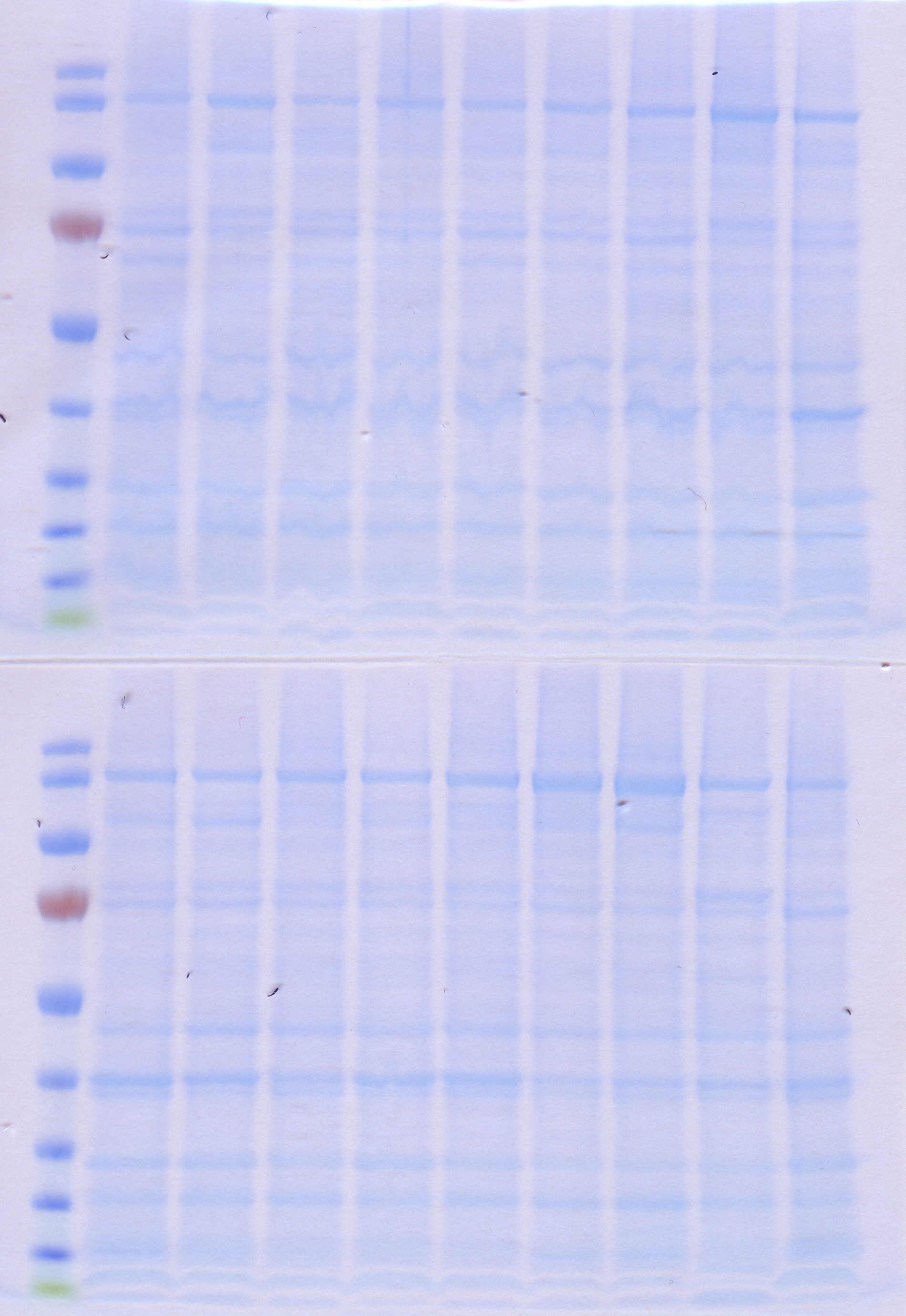

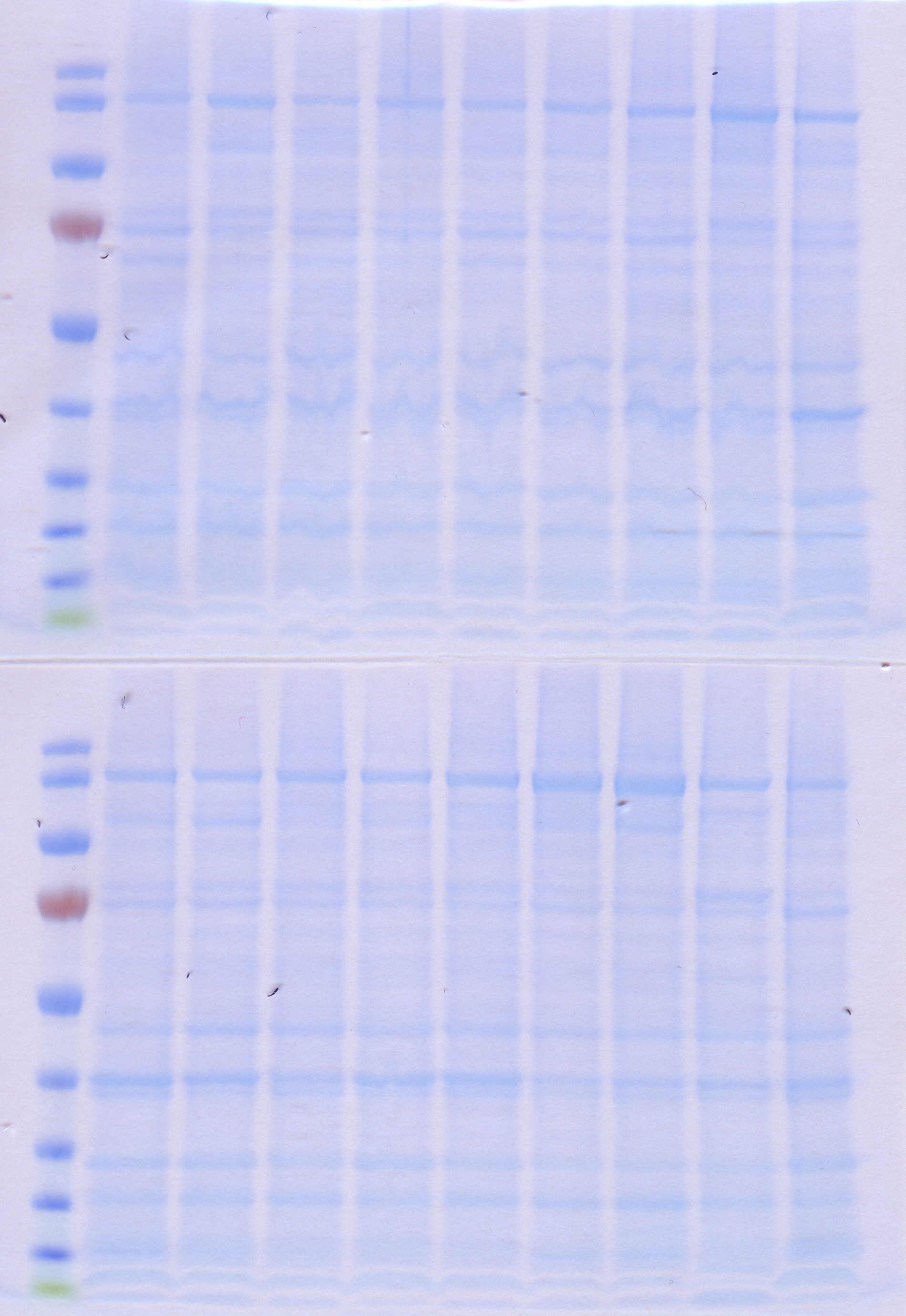


**A_sub_**

**T**

**R**

**B**

**[kDa]**

**140**

**115**

**80**

**65**

**50**

**40**

**30**

**25**

**15**

**10**

**W978A**

**P904A**

**R870A**

**WT**

**Y542A**

**G592A**

**S595A**

**G691A**

**G694A**

**G697A**

**G755A**

**F787A**

**G843A**

**Y844A**

**K848A**

**R870A**

**G882A**

**N885A**

**P904A**

**A922G**

**W978A**


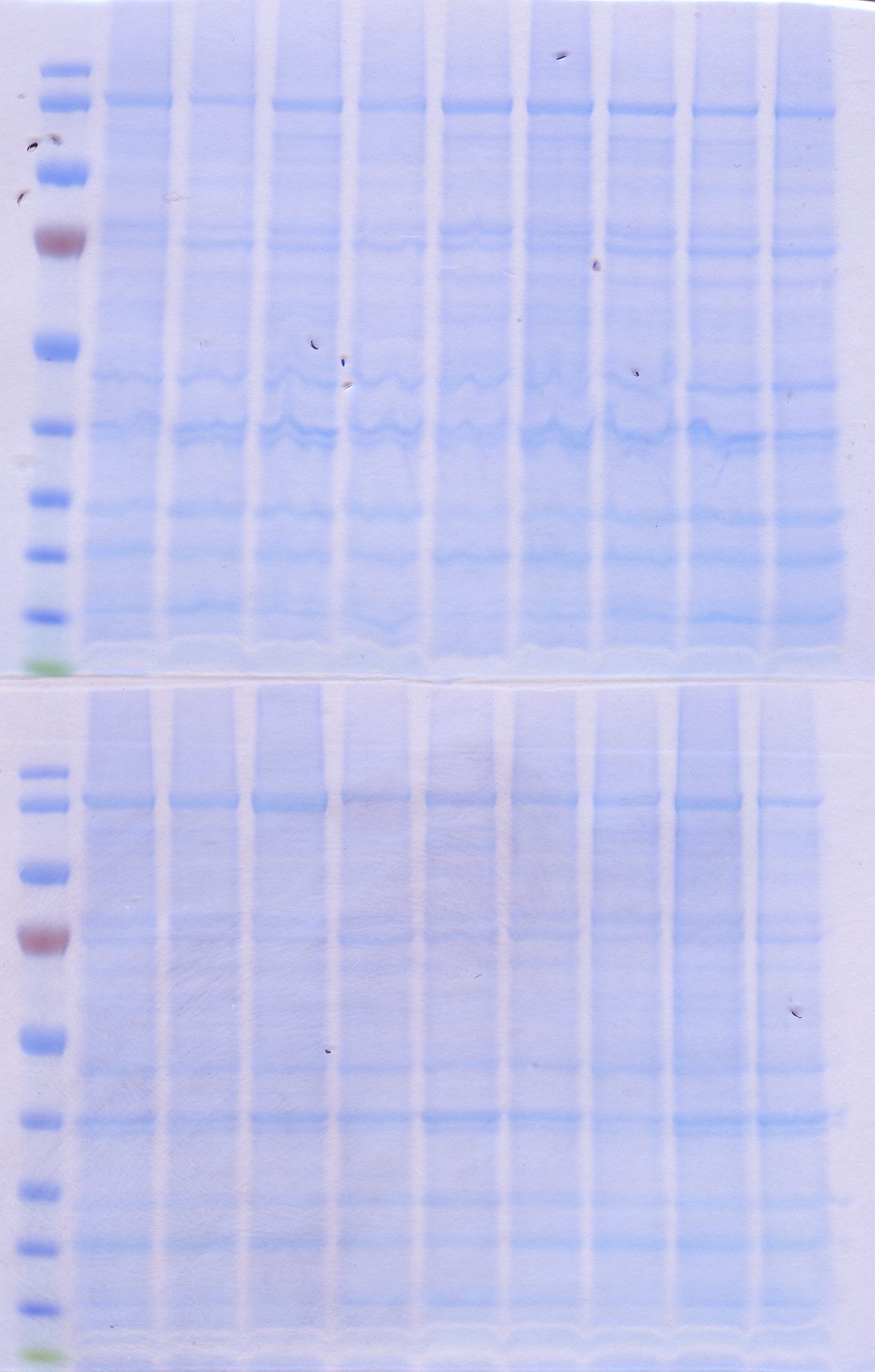

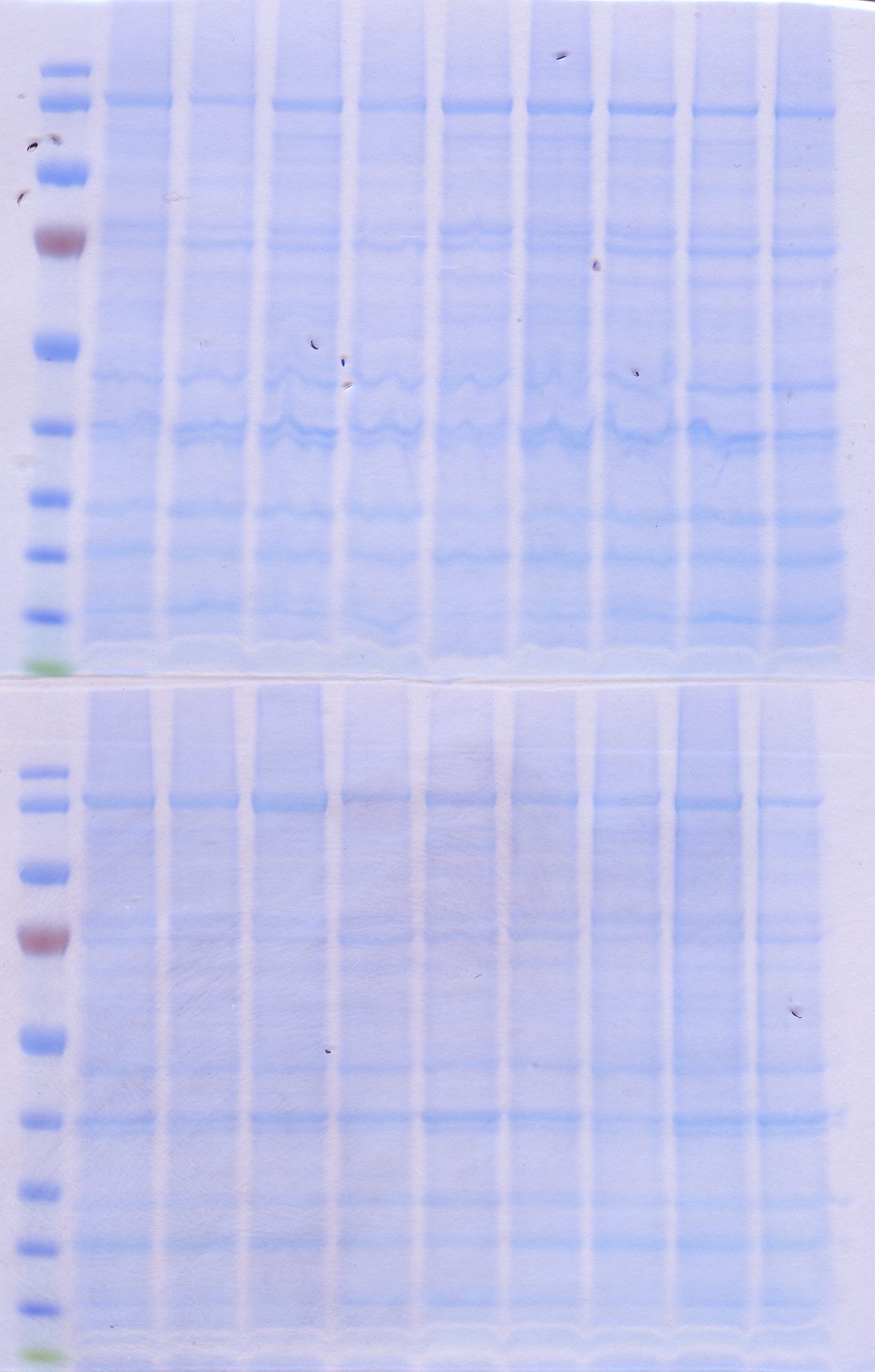


Figure S 3. SDS-PAGE of insoluble fractions (IFs) of *Nc*CAR wild-type and variant enzymes.

IFs were prepared by dissolving the cell pellet, which was obtained after sonication and centrifugation for 1 h at 20,000 x g and 4°C, in 6 M urea. (**A**) Conserved amino acid residues located in the A_core_- and A_sub_-domain exchanged for alanine. Location is indicated by a blue (A_core_) or orange (A_sub_) bar. (**B**) Conserved residues located in the A_sub_-, T-, and R-domain substituted with alanine. Location is indicated by an orange (A_sub_), magenta (T), or green (R) bar. 10 µg of protein were loaded, respectively. The expected molecular weight of *Nc*CAR is 120 kDa (Schwendenwein et al., 2016). All variants showed formation of insoluble protein to a moderate and fairly uniform extend, whereas no soluble expression was visible for variant G184A, R870A, and W978A (highlighted in blue and underlined), or low soluble expression for variant P904A (highlighted in pale blue). Ladder: PageRuler^TM^ Prestained Protein Ladder (Thermo Scientific). NuPAGE^TM^ 4-12% Bis-Tris gels (Thermo Scientific) were run for 50 min at 200 V and 120 mA in MOPS buffer and stained with SimplyBlue^TM^ SafeStain (Invitrogen).

Table S 1. Primers used for site-directed mutagenesis.

| **Designation** | **Forward primer** | | **Reverse primer** | |
| --- | --- | --- | --- | --- |
| **S183A** | **M1f** | 5’-G ATG GTG CTG CAT ACC **GC**C GGT TCT ACG GGC TTT C-3’ | **M1r** | 5’-G AAA GCC CGT AGA ACC G**GC** GGT ATG CAG CAC CAT C-3’ |
| **G184A** | **M2f** | 5’-G GTG CTG CAT ACC AGC G**C**T TCT ACG GGC TTT CC-3’ | **M2r** | 5’-GG AAA GCC CGT AGA A**G**C GCT GGT ATG CAG CAC C-3’ |
| **T186A** | **M3f** | 5’-CAT ACC AGC GGT TCT **G**CG GGC TTT CCG AAA CC-3’ | **M3r** | 5’-GG TTT CGG AAA GCC CG**C** AGA ACC GCT GGT ATG-3’ |
| **P189A** | **M4f** | 5’-GGT TCT ACG GGC TTT **G**CG AAA CCG ATT GTG GCG-3’ | **M4r** | 5’-CGC CAC AAT CGG TTT CG**C** AAA GCC CGT AGA ACC-3’ |
| **K190A** | **M5f** | 5’-CT ACG GGC TTT CCG **GC**A CCG ATT GTG GCG-3’ | **M5r** | 5’-CGC CAC AAT CGG T**GC** CGG AAA GCC CGT AG-3’ |
| **P234A** | **M6f** | 5’-CTG ATG CAT CCG ATG **G**CG CTG TTT CAC GCT GCG-3’ | **M6r** | 5’-CGC AGC GTG AAA CAG CG**C** CAT CGG ATG CAT CAG-3’ |
| **H237A** | **M7f** | 5’-CG ATG CCG CTG TTT **GC**C GCT GCG GGC ATG TAT ATT AG-3’ | **M7r** | 5’-CT AAT ATA CAT GCC CGC AGC G**GC** AAA CAG CGG CAT CG-3’ |
| **T336A** | **M8f** | 5’-G TGC AAT CTG ATC TCG GCC **G**CC GAA TTT ACG CCG TTT C-3’ | **M8r** | 5’-G AAA CGG CGT AAA TTC GG**C** GGC CGA GAT CAG ATT GCA C-3’ |
| **E337A** | **M9f** | 5’-GC AAT CTG ATC TCG GCC ACC G**C**A TTT ACG CCG TTT C-3’ | **M9r** | 5’-GG AAA CGG CGT AAA T**G**C GGT GGC CGA GAT CAG ATT GC-3’ |
| **G432A** | **M10f** | 5’-C GTG TTC TCA AAC G**C**C GAA AAA CTG AAT CCG ATT ACC ATC G-3’ | **M10r** | 5’-C GAT GGT AAT CGG ATT CAG TTT TTC G**G**C GTT TGA GAA CAC G-3’ |
| **E433A** | **M11f** | 5’-C GTG TTC TCA AAC GGC G**C**A AAA CTG AAT CCG ATT ACC ATC G-3’ | **M11r** | 5’-C GAT GGT AAT CGG ATT CAG TTT T**G**C GCC GTT TGA GAA CAC G-3’ |
| **F787A** | **M12f** | 5’-CG GTG GAA TCT **GC**C GAA CCG CAC ATC C-3’ | **M12r** | 5’-G GAT GTG CGG TTC G**GC** AGA TTC CAC CG-3’ |
| **G843A** | **M13f** | 5’-G CTG GCC GCC GGT G**CT** TAC GGC CAG-3’ | **M13r** | 5’-CTG GCC GTA **AG**C ACC GGC GGC CAG C-3’ |
| **Y844A** | **M14f** | 5’-C GCC GGT GGC **GC**C GGC CAG TCC AAA CTG-3’ | **M14r** | 5’-CAG TTT GGA CTG GCC G**GC** GCC ACC GGC G-3’ |
| **K848A** | **M15f** | 5’-GGT GGC TAC GGC CAG TCC **GC**A CTG GTG AGC TCT CTG-3’ | **M15r** | 5’-CAG AGA GCT CAC CAG T**GC** GGA CTG GCC GTA GCC ACC-3’ |
| **R870A** | **M16f** | 5’-G CCG ACC GAA GTC GTG **GC**T GTT GGT CAG GTT GCG G-3’ | **M16r** | 5’-C CGC AAC CTG ACC AAC A**GC** CAC GAC TTC GGT CGG C-3’ |
| **N885A** | **M17f** | 5’-CC GAA AAA GGT TAT TGG **GC**T AAA CAA GAA TGG CTG CCG-3’ | **M17r** | 5’-CGG CAG CCA TTC TTG TTT A**GC** CCA ATA ACC TTT TTC GG-3’ |
| **P285A** | **M18f** | 5’-GGT ATG ATT CTG CCG **G**CG GCT ATC CTG GAA G-3’ | **M18r** | 5’-C TTC CAG GAT AGC CG**C** CGG CAG AAT CAT ACC-3’ |
| **G310A** | **M19f** | 5’-T TTT GTG AGC TTC G**C**C GGT GGC AAC CTG G-3’ | **M19r** | 5’-C CAG GTT GCC ACC G**G**C GAA GCT CAC AAA A-3’ |
| **D405A** | **M20f** | 5’-CG GAA TAT TCC ACG AAA G**C**C CTG TAC AAA CGT CAT CC-3’ | **M20r** | 5’-GG ATG ACG TTT GTA CAG G**G**C TTT CGT GGA ATA TTC CG-3’ |
| **R422A** | **M21f** | 5’-CAC GAA GAT TTT TGG ATT TAT CAG GGT **GC**C GCG GAC AAT ATT A-3’ | **M21r** | 5’-T AAT ATT GTC CGC G**GC** ACC CTG ATA AAT CCA AAA ATC TTC GTG-3’ |
| **E441A** | **M22f** | 5’-G AAT CCG ATT ACC ATC G**C**A GAA ACG CTG CAG GGT C-3’ | **M22r** | 5’-G ACC CTG CAG CGT TTC T**G**C GAT GGT AAT CGG ATT C-3’ |
| **G457A** | **M23f** | 5’-GT GCA GTG GTT GTC G**C**C ACG AAC CGT TTT CA-3’ | **M23r** | 5’-TG AAA ACG GTT CGT G**G**C GAC AAC CAC TGC AC-3’ |
| **Y542A** | **M24f** | 5’-C ATG TAC AAA GCT GAA ATC GAT AAA ATC **GC**C GAA GAC GCA GAA AAA GGT-3’ | **M24r** | 5’-ACC TTT TTC TGC GTC TTC G**GC** GAT TTT ATC GAT TTC AGC TTT GTA CAT G-3’ |
| **G592A** | **M25f** | 5’-GAC TTT TTC ACG GCC G**C**C GTT GAT AGT ATG CAG-3’ | **M25r** | 5’-CTG CAT ACT ATC AAC G**G**C GGC CGT GAA AAA GTC-3’ |
| **S595A** | **M26f** | 5’-C ACG GCC GGC GTT GAT **GC**T ATG CAG GTC ATT ACC-3’ | **M26r** | 5’-GGT AAT GAC CTG CAT A**GC** ATC AAC GCC GGC CGT G-3’ |
| **G691A** | **M27f** | 5’-C GTG GTT ATT ACC G**C**T ACC ACG GGT GGC A-3’ | **M27r** | 5’-T GCC ACC CGT GGT A**G**C GGT AAT AAC CAC G-3’ |
| **G694A** | **M28f** | 5’-CC GGT ACC ACG G**C**T GGC ATC GGC TC-3’ | **M28r** | 5’-GA GCC GAT GCC A**G**C CGT GGT ACC GG-3’ |
| **G697A** | **M29f** | 5’-C ACG GGT GGC ATC G**C**C TCC TAC CTG ATT G-3’ | **M29r** | 5’-C AAT CAG GTA GGA G**G**C GAT GCC ACC CGT G-3’ |
| **G755A** | **M30f** | 5’-C GCT GAC CTG G**C**T CTG GGT CCG G-3’ | **M30r** | 5’-C CGG ACC CAG A**G**C CAG GTC AGC G-3’ |
| **G882A** | **M31f** | 5’-G GGT CCG AGT TCC GAA AAA G**C**T TAT TGG AAT AAA CAA GAA TG-3’ | **M31r** | 5’-CA TTC TTG TTT ATT CCA ATA A**G**C TTT TTC GGA ACT CGG ACC C-3’ |
| **P904A** | **M32f** | 5’-CTG GGT GTG CTG **G**CG GAT AGC CTG G-3’ | **M32r** | 5’-C CAG GCT ATC CG**C** CAG CAC ACC CAG-3’ |
| **A922G** | **M33f** | 5’-CCG ATT GAA GCC ATC G**G**A AAA CTG CTG CTG GAA-3’ | **M33r** | 5’-TTC CAG CAG CAG TTT T**C**C GAT GGC TTC AAT CGG-3’ |
| **W978A** | **M34f** | 5’-TC GTT CCG CTG GAC GAA **GC**G CTG GAA GCG C-3’ | **M34r** | 5’-G CGC TTC CAG C**GC** TTC GTC CAG CGG AAC GA-3’ |
